# Supplementary material for: Hybrid Models and Biological Model Reduction with PyDSTool
Source: PLoS Comput Biol. 2012 Aug 9;8(8):e1002628. doi: 10.1371/journal.pcbi.1002628 (PMC3415397; doi:10.1371/journal.pcbi.1002628)
Supplement: Text S4 — Complete source code for the PyDSTool package (version 0.88.120504). Includes API documentation and help files linking to web pages. This file is identical to the current public release on Sourceforge.net. (ZIP) [file pcbi.1002628.s004.zip › PyDSTool/html/PyDSTool.parseUtils-pysrc.html]

xml version="1.0" encoding="ascii"?


PyDSTool.parseUtils


| Home | Trees | Indices | Help | PyDSTool |
| --- | --- | --- | --- | --- |

Package PyDSTool ::
Module parseUtils

[hide private]

[frames] | no frames]

# Source Code for Module PyDSTool.parseUtils

```
   1  """
 
   2      Parser utilities.
 
   3  
 
   4      Robert Clewley, September 2005.
 
   5  
 
   6      Includes AST code by Pearu Peterson and Ryan Gutenkunst,
 
   7        modified by R. Clewley.
 
   8  """ 
   9  
 
  10  # IMPORTS
 
  11  from __future__ import division 
  12  from errors import * 
  13  from common import * 
  14  import re 
  15  import math, random 
  16  from numpy import alltrue, sometrue 
  17  from copy import copy, deepcopy 
  18  import parser, symbol, token 
  19  
 
  20  # --------------------------------------------------------------------------
 
  21  # GLOBAL BEHAVIOUR CONTROLS -- leave both as True for use with PyDSTool.
 
  22  # switch to change output from using 'x**y' (DO_POW=False)
 
  23  # to 'pow(x,y)' (DO_POW=TRUE)
 
  24  DO_POW=True 
  25  # switch to activate treatment of integer constants as decimals in expressions
 
  26  # and simplifications of expressions.
 
  27  DO_DEC=True 
  28  
 
  29  # ------------------------------------------------------------------------
 
  30  ### Protected names
 
  31  
 
  32  protected_scipynames = ['sign', 'mod'] 
  33  specialfns = ['airy', 'airye', 'ai_zeros', 'bi_zeros', 'ellipj',
 
  34              'ellipk', 'ellipkinc', 'ellipe', 'ellipeinc', 'jn',
 
  35              'jv', 'jve', 'yn', 'yv', 'yve', 'kn', 'kv', 'kve',
 
  36              'iv', 'ive', 'hankel1', 'hankel1e', 'hankel2',
 
  37              'hankel2e', 'lmbda', 'jnjnp_zeros', 'jnyn_zeros',
 
  38              'jn_zeros', 'jnp_zeros', 'yn_zeros', 'ynp_zeros',
 
  39              'y0_zeros', 'y1_zeros', 'y1p_zeros', 'j0', 'j1',
 
  40              'y0', 'y1', 'i0', 'i0e', 'i1', 'i1e', 'k0', 'k0e',
 
  41              'k1', 'k1e', 'itj0y0', 'it2j0y0', 'iti0k0', 'it2i0k0',
 
  42              'besselpoly', 'jvp', 'yvp', 'kvp', 'ivp', 'h1vp',
 
  43              'h2vp', 'sph_jn', 'sph_yn', 'sph_jnyn', 'sph_in',
 
  44              'sph_kn', 'sph_inkn', 'riccati_jn', 'riccati_yn',
 
  45              'struve', 'modstruve', 'itstruve0', 'it2struve0',
 
  46              'itmodstruve0', 'bdtr', 'bdtrc', 'bdtri', 'btdtr',
 
  47              'btdtri', 'fdtr', 'fdtrc', 'fdtri', 'gdtr', 'gdtrc',
 
  48              'gdtria', 'nbdtr', 'nbdtrc', 'nbdtri', 'pdtr', 'pdtrc',
 
  49              'pdtri', 'stdtr', 'stdtridf', 'stdtrit', 'chdtr', 'chdtrc',
 
  50              'chdtri', 'ndtr', 'ndtri', 'smirnov', 'smirnovi',
 
  51              'kolmogorov', 'kolmogi', 'tklmbda', 'gamma',
 
  52              'gammaln', 'gammainc', 'gammaincinv', 'gammaincc',
 
  53              'gammainccinv', 'beta', 'betaln', 'betainc',
 
  54              'betaincinv', 'psi',
 
  55              'digamma', 'rgamma', 'polygamma', 'erf', 'erfc',
 
  56              'erfinv', 'erfcinv', 'erf_zeros', 'fresnel',
 
  57              'fresnel_zeros', 'fresnelc_zeros', 'fresnels_zeros',
 
  58              'modfresnelp', 'modfresnelm', 'lpn', 'lqn', 'lpmn',
 
  59              'lqmn', 'lpmv', 'sph_harm', 'legendre', 'chebyt',
 
  60              'chebyu', 'chebyc', 'chebys', 'jacobi', 'laguerre',
 
  61              'genlaguerre', 'hermite', 'hermitenorm', 'gegenbauer',
 
  62              'sh_legendre', 'sh_chebyt', 'sh_chebyu', 'sh_jacobi',
 
  63              'hyp2f1', 'hyp1f1', 'hyperu', 'hyp0f1', 'hyp2f0',
 
  64              'hyp1f2', 'hyp3f0', 'pbdv', 'pbvv', 'pbwa', 'pbdv_seq',
 
  65              'pbvv_seq', 'pbdn_seq', 'mathieu_a', 'mathieu_b',
 
  66              'mathieu_even_coef', 'mathieu_odd_coef', 'mathieu_cem',
 
  67              'mathieu_sem', 'mathieu_modcem1', 'mathieu_modcem2',
 
  68              'mathieu_modsem1', 'mathieu_modsem2', 'pro_ang1',
 
  69              'pro_rad1', 'pro_rad2', 'obl_ang1', 'obl_rad1',
 
  70              'obl_rad2', 'pro_cv', 'obl_cv', 'pro_cv_seq',
 
  71              'obl_cv_seq', 'pro_ang1_cv', 'pro_rad1_cv',
 
  72              'pro_rad2_cv', 'obl_ang1_cv', 'obl_rad1_cv',
 
  73              'obl_rad2_cv', 'kelvin', 'kelvin_zeros', 'ber',
 
  74              'bei', 'berp', 'beip', 'ker', 'kei', 'kerp', 'keip',
 
  75              'ber_zeros', 'bei_zeros', 'berp_zeros', 'beip_zeros',
 
  76              'ker_zeros', 'kei_zeros', 'kerp_zeros', 'keip_zeros',
 
  77              'expn', 'exp1', 'expi', 'wofz', 'dawsn', 'shichi',
 
  78              'sici', 'spence', 'zeta', 'zetac', 'cbrt', 'exp10',
 
  79              'exp2', 'radian', 'cosdg', 'sindg', 'tandg', 'cotdg',
 
  80              'log1p', 'expm1', 'cosm1', 'round'] 
  81  protected_specialfns = ['special_'+s for s in specialfns] 
  82  protected_mathnames = filter(lambda s: not s.startswith('__'), \
 
  83                                   dir(math)) 
  84  protected_randomnames = filter(lambda s: not s.startswith('_'), \
 
  85                                   dir(random))  # yes, just single _ 
  86  # We add internal default auxiliary function names for use by
 
  87  # functional specifications.
 
  88  builtin_auxnames = ['globalindepvar', 'initcond', 'heav', 'if',
 
  89                      'getindex', 'getbound'] 
  90  
 
  91  protected_macronames = ['for', 'if', 'max', 'min', 'sum'] 
  92  
 
  93  reserved_keywords = ['and', 'not', 'or', 'del', 'for', 'if', 'is', 'raise',
 
  94                  'assert', 'elif', 'from', 'lambda', 'return', 'break', 'else',
 
  95                  'global', 'try', 'class', 'except', 'while',
 
  96                  'continue', 'exec', 'import', 'pass', 'yield', 'def',
 
  97                  'finally', 'in', 'print', 'as', 'None'] 
  98  
 
  99  convert_power_reserved_keywords = ['del', 'for', 'if', 'is', 'raise',
 
 100                  'assert', 'elif', 'from', 'lambda', 'return', 'break', 'else',
 
 101                  'global', 'try', 'class', 'except', 'while',
 
 102                  'continue', 'exec', 'import', 'pass', 'yield', 'def',
 
 103                  'finally', 'in', 'print', 'as', 'None'] 
 104  
 
 105  # 'abs' is defined in python core, so doesn't appear in math
 
 106  protected_allnames = protected_mathnames + protected_scipynames \
 
 107                      + protected_specialfns + protected_randomnames \
 
 108                      + builtin_auxnames + protected_macronames \
 
 109                      + ['abs', 'pow', 'min', 'max', 'sum'] 
 110  
 
 111  # signature lengths for builtin auxiliary functions and macros, for
 
 112  # use by ModelSpec in eval() method (to create correct-signatured temporary
 
 113  # functions).
 
 114  builtinFnSigInfo = {'globalindepvar': 1, 'initcond': 1, 'heav': 1, 'getindex': 1,
 
 115                      'if': 3, 'for': 4, 'getbound': 2, 'max': 1, 'min': 1} 
 116  
 
 117  # ------------------------------------------------------------------------
 
 118  
 
 119  # EXPORTS
 
 120  _functions = ['readArgs', 'findEndBrace', 'makeParList', 'joinStrs',
 
 121                'parseMatrixStrToDictStr', 'joinAsStrs', 'replaceSep',
 
 122                'wrapArgInCall', 'addArgToCalls', 'findNumTailPos',
 
 123                'isToken', 'isNameToken', 'isNumericToken', 'count_sep',
 
 124                'isHierarchicalName', 'replaceSepInv', 'replaceSepListInv',
 
 125                'replaceSepList', 'convertPowers', 'mapNames',
 
 126                'replaceCallsWithDummies', 'isIntegerToken', 'proper_match',
 
 127                'remove_indices_from_range'] 
 128  
 
 129  _objects = ['protected_auxnamesDB', 'protected_allnames', 'protected_macronames',
 
 130              'protected_mathnames', 'protected_randomnames', 'builtin_auxnames',
 
 131              'protected_scipynames', 'protected_specialfns', 'builtinFnSigInfo'] 
 132  
 
 133  _classes = ['symbolMapClass', 'parserObject', 'auxfnDBclass'] 
 134  
 
 135  _constants = ['name_chars_RE', 'num_chars', 'ZEROS', 'ONES', 'NAMESEP',
 
 136                '_indentstr'] 
 137  
 
 138  _symbfuncs = ['simplify', 'simplify_str', 'ensurebare', 'ensureparen',
 
 139               'trysimple', 'ensuredecimalconst', 'doneg', 'dosub', 'doadd',
 
 140               'dodiv', 'domul', 'dopower', 'splitastLR', 'ast2string', 'string2ast',
 
 141               'sym2name', 'ast2shortlist', 'splitargs', 'mapPowStr',
 
 142               'toPowSyntax', 'ensureparen_div'] 
 143  
 
 144  _symbconsts = ['syms'] 
 145  
 
 146  __all__ = _functions + _classes + _objects + _constants + _symbfuncs + _symbconsts 
 147  
 
 148  #-----------------------------------------------------------------------------
 
 149  
 
 150  ## constants for parsing
 
 151  name_chars_RE = re.compile('\w') 
 152  alphabet_chars_RE = re.compile('[a-zA-Z0-9]')   # without the '_' 
 153  num_chars = map(lambda i: str(i), range(10)) 
 154  
 
 155  if DO_POW: 
 156      POW_STR = 'pow(%s,%s)' 
 157  else: 
 158      POW_STR = '%s**%s' 
 159  
 
 160  if DO_DEC: 
 161      ZEROS = ['0','0.0','0.','(0)','(0.0)','(0.)'] 
 162      ONES = ['1','1.0','1.','(1)','(1.0)','(1.)'] 
 163      TENS = ['10', '10.0', '10.','(10)','(10.0)','(10.)'] 
 164  else: 
 165      ZEROS = ['0'] 
 166      ONES = ['1'] 
 167      TENS = ['10'] 
 168  
 
 169  # separator for compound, hierarchical names
 
 170  NAMESEP = '.' 
 171  
 
 172  # for python indentation in automatically generated code, use 4 spaces
 
 173  _indentstr = "    " 
 174  
 
 175  
 
 176  # ----------------------------------------------------------------------------
 
 177  # This section: code by Pearu Peterson, adapted by Ryan Gutenkunst
 
 178  #   and Robert Clewley.
 
 179  
 
 180  syms=token.tok_name 
 181  for s in symbol.sym_name.keys(): 
 182      syms[s]=symbol.sym_name[s] 
 183  
 
 184  
 


185 -def mapPowStr(t, p='**'):


186      """Input an expression of the form 'pow(x,y)'. Outputs an expression of the
 
 187      form x**y, x^y, or pow(x,y) and where x and y have also been processed to the
 
 188      target power syntax.
 
 189      Written by R. Clewley""" 
 190      ll = splitargs(ast2string(t[2])[1:-1]) 
 191      if p=='**': 
 192          lpart = dopower(ensureparen(ast2string(toDoubleStarSyntax(string2ast(ll[0]))),1),
 
 193                         ensureparen(ast2string(toDoubleStarSyntax(string2ast(ll[1]))),1),
 
 194                         '%s**%s') 
 195          if len(t) > 3: 
 196              res = ensureparen(ast2string(toDoubleStarSyntax(['power',string2ast('__LPART__')]+t[3:]))) 
 197              return res.replace('__LPART__', lpart) 
 198          else: 
 199              return ensureparen(lpart,1) 
 200      elif p=='^': 
 201          lpart = dopower(ensureparen(ast2string(toCircumflexSyntax(string2ast(ll[0]))),1),
 
 202                         ensureparen(ast2string(toCircumflexSyntax(string2ast(ll[1]))),1),
 
 203                         '%s^%s') 
 204          if len(t) > 3: 
 205              res = ensureparen(ast2string(toCircumflexSyntax(['power',string2ast('__LPART__')]+t[3:])),1) 
 206              return res.replace('__LPART__', lpart) 
 207          else: 
 208              return ensureparen(lpart,1) 
 209      elif p=='pow': 
 210          lpart = dopower(ensurebare(ast2string(toPowSyntax(string2ast(ll[0])))),
 
 211                         ensurebare(ast2string(toPowSyntax(string2ast(ll[1])))),
 
 212                         'pow(%s,%s)') 
 213          if len(t) > 3: 
 214              res = ensurebare(ast2string(toPowSyntax(['power',string2ast('__LPART__')]+t[3:]))) 
 215              return res.replace('__LPART__', lpart) 
 216          else: 
 217              return ensureparen(lpart,1) 
 218      else: 
 219          raise ValueError("Invalid power operator")

 220  
 
 221  
 


222 -def toCircumflexSyntax(t):


223      # R. Clewley
 
 224      if isinstance(t[0], str): 
 225          if t[0] == 'power': 
 226              if t[2][0] == 'DOUBLESTAR': 
 227                  return string2ast(ensureparen(dopower(ast2string(toCircumflexSyntax(t[1])),
 
 228                                      ast2string(toCircumflexSyntax(t[3])),
 
 229                           '%s^%s'),1)) 
 230              if t[1] == ['NAME', 'pow']: 
 231                  return string2ast(ensureparen(mapPowStr(t,'^'),1)) 
 232      o = [] 
 233      for i in t: 
 234          if isinstance(i,list): 
 235              if type(i[0]) == str and i[0].islower(): 
 236                  o.append(toCircumflexSyntax(i)) 
 237              else: 
 238                  o.append(i) 
 239          else: 
 240              o.append(i) 
 241      return o

 242  
 
 243  
 


244 -def toDoubleStarSyntax(t):


245      # R. Clewley
 
 246      if isinstance(t[0], str): 
 247          if t[0] == 'xor_expr' and t[2][0]=='CIRCUMFLEX': 
 248              # ^ syntax has complex binding rules in python parser's AST!
 
 249              # trick - easy to convert to ** first. then, using a bit of a hack
 
 250              # convert to string and back to AST so that proper AST for **
 
 251              # is formed.
 
 252              tc = copy(t) 
 253              tc[0] = 'power' 
 254              tc[2] = ['DOUBLESTAR', '**'] 
 255              return toDoubleStarSyntax(string2ast(ast2string(tc)))   # yes, i mean this 
 256          if t[0] == 'power' and t[1] == ['NAME', 'pow']: 
 257              return string2ast(ensureparen(mapPowStr(t,'**'),1)) 
 258      o = [] 
 259      for i in t: 
 260          if isinstance(i,list): 
 261              if type(i[0]) == str and i[0].islower(): 
 262                  o.append(toDoubleStarSyntax(i)) 
 263              else: 
 264                  o.append(i) 
 265          else: 
 266              o.append(i) 
 267      return o

 268  
 
 269  
 


270 -def toPowSyntax(t):


271      # R. Clewley
 
 272      if isinstance(t[0],str): 
 273          if t[0] == 'power': 
 274              try: 
 275                  if t[2][0]=='DOUBLESTAR': 
 276                      try: 
 277                          return string2ast(dopower(ensurebare(ast2string(toPowSyntax(t[1]))),
 
 278                                              ensurebare(ast2string(toPowSyntax(t[3]))),
 
 279                                   'pow(%s,%s)')) 
 280                      except IndexError: 
 281                          # there's a pow statement already here, not a **
 
 282                          # so ignore
 
 283                          return t 
 284                  elif t[1][1] == 'pow': 
 285                      return string2ast(ensureparen(mapPowStr(t,'pow'),1)) 
 286                  elif len(t)>3 and t[3][0]=='DOUBLESTAR': 
 287                      try: 
 288                          return string2ast(dopower(ensurebare(ast2string(toPowSyntax(t[1:3]))),
 
 289                                              ensurebare(ast2string(toPowSyntax(t[4]))),
 
 290                                   'pow(%s,%s)')) 
 291                      except IndexError: 
 292                          # there's a pow statement already here, not a **
 
 293                          # so ignore
 
 294                          return t 
 295              except: 
 296                  print t 
 297                  print ast2string(t) 
 298                  raise 
 299          elif t[0] == 'xor_expr' and t[2][0]=='CIRCUMFLEX': 
 300              # ^ syntax has complex binding rules in python parser's AST!
 
 301              # trick - easy to convert to ** first. then, using a bit of a hack
 
 302              # convert to string and back to AST so that proper AST for **
 
 303              # is formed.
 
 304              tc = copy(t) 
 305              tc[0] = 'power' 
 306              tc[2] = ['DOUBLESTAR', '**'] 
 307              return toPowSyntax(string2ast(ast2string(tc)))   # yes, i mean this 
 308      o = [] 
 309      for i in t: 
 310          if isinstance(i,list): 
 311              if type(i[0]) == str and i[0].islower(): 
 312                  o.append(toPowSyntax(i)) 
 313              else: 
 314                  o.append(i) 
 315          else: 
 316              o.append(i) 
 317      return o

 318  
 
 319  
 


320 -def temp_macro_names(s):


321      t = s 
 322      for m in convert_power_reserved_keywords: 
 323          t = t.replace(m, '__'+m+'__') 
 324      return t

 325  
 


326 -def temp_macro_names_inv(s):


327      t = s 
 328      for m in convert_power_reserved_keywords: 
 329          t = t.replace('__'+m+'__', m) 
 330      return t

 331  
 
 332  
 


333 -def convertPowers(s, target="pow"):


334      """convertPowers takes a string argument and maps all occurrences
 
 335      of power sub-expressions into the chosen target syntax. That option
 
 336      is one of "**", "^", or "pow".""" 
 337      # temp_macro_names switches python reserved keywords to adapted names
 
 338      # that won't make the python parser used in string2ast raise a syntax error
 
 339      # ... but only invoke this relatively expensive function in the unlikely
 
 340      # event a clash occurs.
 
 341      # R. Clewley
 
 342      if target=="**": 
 343          try: 
 344              return ast2string(toDoubleStarSyntax(string2ast(s))) 
 345          except SyntaxError: 
 346              s = temp_macro_names(s) 
 347              return temp_macro_names_inv(ast2string(toDoubleStarSyntax(string2ast(s)))) 
 348      elif target=="^": 
 349          try: 
 350              return ast2string(ensureints(toCircumflexSyntax(string2ast(s)))) 
 351          except SyntaxError: 
 352              s = temp_macro_names(s) 
 353              return temp_macro_names_inv(ast2string(ensureints(toCircumflexSyntax(string2ast(s))))) 
 354      elif target=="pow": 
 355          try: 
 356              return ast2string(toPowSyntax(string2ast(s))) 
 357          except SyntaxError: 
 358              s = temp_macro_names(s) 
 359              return temp_macro_names_inv(ast2string(toPowSyntax(string2ast(s)))) 
 360      else: 
 361          raise ValueError("Invalid target syntax")

 362  
 
 363  
 


364 -def ensureints(t):


365      """Ensure that any floating point constants appearing in t that are
 
 366      round numbers get converted to integer representations.""" 
 367      if type(t)==str: 
 368          return ast2string(ensureints(string2ast(t))) 
 369      o = [] 
 370      for i in t: 
 371          if type(i) == list: 
 372              if type(i[0]) == str and i[0].islower(): 
 373                  o.append(ensureints(i)) 
 374              elif i[0]=='NUMBER': 
 375                  # CAPS for constants
 
 376                  o.append(string2ast(trysimple(ast2string(i)))) 
 377              else: 
 378                  o.append(i) 
 379          else: 
 380              o.append(i) 
 381      return o

 382  
 
 383  
 


384 -def splitargs(da, lbraces=['('], rbraces=[')']):


385      """Function to split string-delimited arguments in a string without
 
 386      being fooled by those that occur in function calls.
 
 387      Written by Pearu Peterson. Adapted by Rob Clewley to accept different
 
 388      braces.""" 
 389      if alltrue([da.find(lbrace)<0 for lbrace in lbraces]): 
 390          return da.split(',') 
 391      ll=[];o='';ii=0 
 392      for i in da: 
 393          if i==',' and ii==0: 
 394              ll.append(o) 
 395              o='' 
 396          else: 
 397              if i in lbraces: ii=ii+1 
 398              if i in rbraces: ii=ii-1 
 399              o=o+i 
 400      ll.append(o) 
 401      return ll

 402  
 


403 -def ast2shortlist(t):


404      if type(t) is parser.ASTType: return ast2shortlist(t.tolist()) 
 405      if not isinstance(t, list): return t 
 406      if t[1] == '': return None 
 407      if not isinstance(t[1], list): return t 
 408      if len(t) == 2 and isinstance(t[1], list): 
 409          return ast2shortlist(t[1]) 
 410      o=[] 
 411      for tt in map(ast2shortlist, t[1:]): 
 412          if tt is not None: 
 413              o.append(tt) 
 414      if len(o)==1: return o[0] 
 415      return [t[0]]+o

 416  
 


417 -def sym2name(t):


418      if type(t) is parser.ASTType: return sym2name(t.tolist()) 
 419      if not isinstance(t, list): return t 
 420      return [syms[t[0]]]+map(sym2name,t[1:])

 421  
 


422 -def string2ast(t):


423      return sym2name(ast2shortlist(parser.expr(t)))

 424  
 


425 -def ast2string(t):


426      #if isinstance(t, str): return t
 
 427      if type(t) is parser.ASTType: return ast2string(t.tolist()) 
 428      if not isinstance(t, list): return None 
 429      if not isinstance(t[1], list): return t[1] 
 430      o='' 
 431      for tt in map(ast2string,t): 
 432          if isinstance(tt, str): 
 433              o=o+tt 
 434      return o

 435  
 


436 -def splitastLR(t):


437      lft=t[1] 
 438      rt=t[3:] 
 439      if len(rt)>1: 
 440          rt=[t[0]]+rt 
 441      else: 
 442          rt=rt[0] 
 443      return lft,rt

 444  
 


445 -def dopower(l,r,pow_str=POW_STR):


446      if r in ZEROS: return '1' 
 447      if l in ZEROS: return '0' 
 448      if l in ONES: return '1' 
 449      if r in ONES: return l 
 450      if pow_str=='%s**%s': 
 451          return trysimple('%s**%s'%(ensureparen(l),ensureparen(r))) 
 452      elif pow_str == '%s^%s': 
 453          return trysimple('%s^%s'%(ensuredecimalconst(ensureparen(l)),ensuredecimalconst(ensureparen(r)))) 
 454      elif pow_str == 'pow(%s,%s)': 
 455          return trysimple('pow(%s,%s)'%(l,r)) #trysimple('pow(%s,%s)'%(ensurebare(l),ensurebare(r))) 
 456      else: 
 457          raise ValueError("Invalid target power syntax")

 458  
 


459 -def domul(l,r):


460      if l in ZEROS or r in ZEROS: return '0' 
 461      if l in ONES: return r 
 462      if r in ONES: return l 
 463      if l in ['-'+o for o in ONES]: return doneg(r) 
 464      if r in ['-'+o for o in ONES]: return doneg(l) 
 465      lft = string2ast(l) 
 466      rt = string2ast(r) 
 467      lft_neg = lft[0] == 'factor' and lft[1][0]=='MINUS' 
 468      rt_neg = rt[0] == 'factor' and rt[1][0]=='MINUS' 
 469      if lft_neg: 
 470          new_l = l[1:] 
 471      else: 
 472          new_l = l 
 473      if rt_neg: 
 474          new_r = r[1:] 
 475      else: 
 476          new_r = r 
 477      if lft_neg and rt_neg or not (lft_neg or rt_neg): 
 478          return trysimple('%s*%s'%(ensureparen(new_l,ismul=1),
 
 479                                    ensureparen(new_r,ismul=1))) 
 480      else: 
 481          return trysimple('-%s*%s'%(ensureparen(new_l,ismul=1),
 
 482                                     ensureparen(new_r,ismul=1)))

 483  
 


484 -def dodiv(l,r):


485      if r in ZEROS: raise ValueError("Division by zero in expression") 
 486      if l in ZEROS: return '0' 
 487      if r in ONES: return l 
 488  ##    if l in ['-'+o for o in ONES]: return doneg(dodiv('1',r))
 
 489      if r in ['-'+o for o in ONES]: return doneg(l) 
 490      if r==l: return '1' 
 491      lft = string2ast(l) 
 492      rt = string2ast(r) 
 493      lft_neg = lft[0] == 'factor' and lft[1][0]=='MINUS' 
 494      rt_neg = rt[0] == 'factor' and rt[1][0]=='MINUS' 
 495      if lft_neg: 
 496          new_l = l[1:] 
 497      else: 
 498          new_l = l 
 499      if rt_neg: 
 500          new_r = r[1:] 
 501      else: 
 502          new_r = r 
 503      if lft_neg and rt_neg or not (lft_neg or rt_neg): 
 504          return trysimple('%s/%s'%(ensureparen(ensuredecimalconst(new_l),ismul=1,do_decimal=DO_DEC),
 
 505                                 ensureparen(ensuredecimalconst(new_r),1,do_decimal=DO_DEC)),
 
 506                           do_decimal=DO_DEC) 
 507      else: 
 508          return trysimple('-%s/%s'%(ensureparen(ensuredecimalconst(new_l),ismul=1,do_decimal=DO_DEC),
 
 509                                 ensureparen(ensuredecimalconst(new_r),1,do_decimal=DO_DEC)),
 
 510                           do_decimal=DO_DEC)

 511  
 


512 -def doadd(l,r):


513      if l in ZEROS and r in ZEROS: return '0' 
 514      if l in ZEROS: return r 
 515      if r in ZEROS: return l 
 516      if l==r: return trysimple(domul('2',l)) 
 517      if r[0]=='-': return trysimple('%s%s'%(l,r)) 
 518      return trysimple('%s+%s'%(l,r))

 519  
 


520 -def dosub(l,r):


521      if l in ZEROS and r in ZEROS: return '0' 
 522      if l in ZEROS: return doneg(r) 
 523      if r in ZEROS: return l 
 524      if l==r: return '0' 
 525      if r[0]=='-': return ensureparen(trysimple('%s+%s'%(l,doneg(r)))) 
 526      return trysimple('%s-%s'%(l,r))

 527  
 


528 -def doneg(l):


529      if l in ZEROS: return '0' 
 530  ##    if l[0]=='-': return l[1:]
 
 531      t=string2ast(l) 
 532  ##    print "doneg called with %s"%l, "\n", t, "\n"
 
 533      if t[0]=='atom' and t[1][0]=='LPAR' and t[-1][0]=='RPAR': 
 534          return ensureparen(doneg(ast2string(t[2]))) 
 535      if t[0]=='arith_expr': 
 536          # Propagate -ve sign into the sum
 
 537          o=doneg(ast2string(t[1])) 
 538          aexpr = t[2:] 
 539          for i in range(0,len(aexpr),2): 
 540              if aexpr[i][0]=='PLUS': 
 541                  o = dosub(o, ast2string(aexpr[i+1])) 
 542              else: 
 543                  o = doadd(o, ast2string(aexpr[i+1])) 
 544          return o 
 545      if t[0]=='term': 
 546          # Propagate -ve sign onto the first term
 
 547          tc = copy(t) 
 548          if t[1][0]=='factor' and t[1][1][0]=='MINUS': 
 549              tc[1] = t[1][2] 
 550          else: 
 551              tc[1] = string2ast(doneg(ast2string(t[1]))) 
 552          return ast2string(tc) 
 553      if t[0]=='factor' and t[1][0] == 'MINUS': 
 554          return ast2string(t[2]) 
 555      return trysimple('-%s'%l)

 556  
 


557 -def ensuredecimalconst(t):


558      return trysimple(t,do_decimal=DO_DEC)

 559  
 


560 -def trysimple(t,do_decimal=False):


561      try: 
 562          t_e = eval(t, {}, {}) 
 563          add_point = do_decimal and t_e != 0 and DO_DEC 
 564          if type(t_e) == int and add_point: 
 565              t = repr(t_e)+".0" 
 566          elif type(t_e) == float and int(t_e)==t_e: 
 567              # explicitly use repr here in case t string was e.g. '2e7'
 
 568              # which evals to a float, but the string itself represents an int
 
 569              if add_point: 
 570                  t = repr(t_e) 
 571              else: 
 572                  t = repr(int(t_e)) 
 573          else: 
 574              t = repr(t_e) 
 575      except: 
 576          pass 
 577      return t

 578  
 


579 -def ensureparen_div(tt):


580      if tt[0] == 'term' and tt[2][0] == 'SLASH' and len(tt[3:])>1: 
 581          return ['term',
 
 582                  string2ast(ensureparen(ast2string(tt[1])+'/'+ast2string(tt[3]),
 
 583                                         1))] + tt[4:] 
 584      else: 
 585          return tt

 586  
 


587 -def ensureparen(t,flag=0,ismul=0,do_decimal=False):


588      t=trysimple(t, do_decimal) 
 589      tt=string2ast(t) 
 590      if t[0]=='-': 
 591          if tt[0] == 'factor': # or tt[0] == 'term' and ismul: 
 592              # single number doesn't need braces
 
 593              return t 
 594          else: 
 595  ##        print "0: ", t, "->", '(%s)'%t
 
 596              return '(%s)'%t 
 597      if tt[0]=='arith_expr': 
 598  ##        print "1: ", t, "->", '(%s)'%t
 
 599          return '(%s)'%t 
 600      if flag>0: 
 601          if tt[0] == 'term': 
 602              return '(%s)'%t 
 603          elif tt[0] == 'power': 
 604              if tt[1] == ['NAME', 'pow']: 
 605                  return t 
 606              else: 
 607                  # ** case
 
 608                  for x in tt[1:]: 
 609                      if x[0] == 'arith_expr': 
 610                          return '(%s)'%t 
 611          elif tt[0] == 'xor_expr':    # added xor_expr for ^ powers 
 612              if len(tt)>3: 
 613                  for x in tt[1:]: 
 614                      if x[0] == 'arith_expr': 
 615                          return '(%s)'%t 
 616              else: 
 617                  return t 
 618  ##        if t[0]=='(' and t[-1]==')' and t[1:-1].find('(') < t[1:-1].find(')'):
 
 619  ##            # e.g. (1+x) doesn't need another set of braces
 
 620  ##            print "2: ", t, "->", t
 
 621  ##            return t
 
 622  ##        else:
 
 623  ##            # e.g. (1+x)-(3-y) does need braces
 
 624  ##            print "3: ", t, "->", '(%s)'%t
 
 625  ##            return '(%s)'%t
 
 626      return t

 627  
 


628 -def ensurebare(t):


629      """Ensure no braces in string expression (where possible).
 
 630      Written by Robert Clewley""" 
 631      t=trysimple(t) 
 632      try: 
 633          if t[0]=='(' and t[-1]==')': 
 634              if t[1:-1].find('(') < t[1:-1].find(')'): 
 635                  return t[1:-1] 
 636              else: 
 637                  # false positive, e.g. (1+x)-(3-y)
 
 638                  return t 
 639          else: 
 640              return t 
 641      except IndexError: 
 642          return t

 643  
 


644 -def collect_numbers(t):


645      """Re-arrange 'term' or 'arith_expr' expressions to combine numbers.
 
 646      Numbers go first unless in a sum the numeric term is negative and not all
 
 647      the remaining terms are negative.""" 
 648      # number may be prefixed by a 'factor'
 
 649  ##    print "collect called with %s"%ast2string(t), "\n", t, "\n"
 
 650      if t[0] == 'arith_expr': 
 651          args = [simplify(a) for a in t[1::2]] 
 652          numargs = len(args) 
 653          if args[0][0] == 'factor' and args[0][1][0] == 'MINUS': 
 654              ops = [-1] 
 655              # remove negative sign from term as we've recorded the sign for the sum
 
 656              args[0] = args[0][2] 
 657          else: 
 658              ops = [1] 
 659          for op_t in t[2::2]: 
 660              if op_t[0]=='PLUS': 
 661                  ops.append(1) 
 662              else: 
 663                  ops.append(-1) 
 664          num_ixs = [] 
 665          oth_ixs = [] 
 666          for i, a in enumerate(args): 
 667              if a[0]=='NUMBER': 
 668                  num_ixs.append(i) 
 669              else: 
 670                  oth_ixs.append(i) 
 671          res_num = '0' 
 672          # enter numbers first
 
 673          for nix in num_ixs: 
 674              if ops[nix] > 0: 
 675                  res_num=doadd(res_num,ast2string(simplify(args[nix]))) 
 676              else: 
 677                  res_num=dosub(res_num,ast2string(simplify(args[nix]))) 
 678          # follow by other terms
 
 679          res_oth = '0' 
 680          for oix in oth_ixs: 
 681              if ops[oix] > 0: 
 682                  res_oth=doadd(res_oth,ast2string(simplify(args[oix]))) 
 683              else: 
 684                  res_oth=dosub(res_oth,ast2string(simplify(args[oix]))) 
 685          if res_num[0] == '-' and res_oth[0] != '-': 
 686              # switch order
 
 687              return string2ast(doadd(res_oth,res_num)) 
 688          else: 
 689              return string2ast(doadd(res_num,res_oth)) 
 690      elif t[0] == 'term': 
 691          args = [simplify(a) for a in t[1::2]] 
 692          numargs = len(args) 
 693          ops = [1] 
 694          for op_t in t[2::2]: 
 695              if op_t[0]=='STAR': 
 696                  ops.append(1) 
 697              else: 
 698                  ops.append(-1) 
 699          num_ixs = [] 
 700          oth_ixs = [] 
 701          for i, a in enumerate(args): 
 702              if a[0]=='NUMBER': 
 703                  num_ixs.append(i) 
 704              else: 
 705                  oth_ixs.append(i) 
 706          res_numerator = '1' 
 707          res_denominator = '1' 
 708          # enter numbers first
 
 709          for nix in num_ixs: 
 710              if ops[nix] > 0: 
 711                  res_numerator=domul(res_numerator,ast2string(simplify(args[nix]))) 
 712              else: 
 713                  res_denominator=domul(res_denominator,ast2string(simplify(args[nix]))) 
 714          # follow by other terms
 
 715          for oix in oth_ixs: 
 716              if ops[oix] > 0: 
 717                  res_numerator=domul(res_numerator,ast2string(simplify(args[oix]))) 
 718              else: 
 719                  res_denominator=domul(res_denominator,ast2string(simplify(args[oix]))) 
 720          return string2ast(dodiv(res_numerator,res_denominator)) 
 721      else: 
 722          return t

 723  
 


724 -def simplify_str(s):


725      return s

 726  ##    """String output version of simplify"""
 
 727  ##    t=string2ast(s)
 
 728  ##    if isinstance(t, list) and len(t) == 1:
 
 729  ##        return ast2string(simplify(t[0]))
 
 730  ##    if t[0]=='NUMBER':
 
 731  ##        if 'e' in s:
 
 732  ##            epos=s.find('e')
 
 733  ##            man=s[:epos]
 
 734  ##            ex=s[epos:]
 
 735  ##        else:
 
 736  ##            ex=''
 
 737  ##            man=s
 
 738  ##        if man[-1] == '.':
 
 739  ##            return man+'0'+ex
 
 740  ##        else:
 
 741  ##            return s
 
 742  ##    if t[0]=='NAME':
 
 743  ##        return s
 
 744  ##    if t[0]=='factor':
 
 745  ##        return doneg(ensureparen(ast2string(simplify(t[2:][0]))))
 
 746  ##    if t[0]=='arith_expr':
 
 747  ##        return ast2string(collect_numbers(t))
 
 748  ##    if t[0]=='term':
 
 749  ##        return ast2string(collect_numbers(ensureparen_div(t)))
 
 750  ##    if t[0]=='power': # covers math functions like sin, cos, and log10
 
 751  ##        if t[2][0]=='trailer':
 
 752  ##            if len(t)>3:
 
 753  ##                term1 = simplify(t[:3])
 
 754  ##                if term1[0] in ['NUMBER', 'NAME']: #'power'
 
 755  ##                    formatstr='%s'
 
 756  ##                else:
 
 757  ##                    formatstr='(%s)'
 
 758  ##                return ast2string([t[0],string2ast(formatstr%ast2string(term1)),
 
 759  ##                                  simplify(t[3:])])
 
 760  ##            elif len(t)==3 and t[1][1]=='pow':
 
 761  ##                # 'pow' syntax case
 
 762  ##                terms = t[2][2][1::2]
 
 763  ##                return dopower(ast2string(simplify(terms[0])),
 
 764  ##                               ast2string(simplify(terms[1])))
 
 765  ##            # else ignore and carry on
 
 766  ##        ts=[];o=[];
 
 767  ##        for i in t[1:]:
 
 768  ##            if i[0]=='DOUBLESTAR':
 
 769  ##                if len(o)==1: o=o[0]
 
 770  ##                ts.append(o);
 
 771  ##                o=[]
 
 772  ##            else: o.append(simplify(i))
 
 773  ##        if len(o)==1: o=o[0]
 
 774  ##        ts.append(o)
 
 775  ##        if t[2][0]=='DOUBLESTAR':
 
 776  ##            st,lft,rt=map(ast2string,[t,ts[0],ts[1]])
 
 777  ##            return dopower(simplify_str(lft),simplify_str(rt))
 
 778  ##        if t[2][0]=='trailer':
 
 779  ##            return ast2string(simplify(ts[0]))
 
 780  ##    if t[0] in ['arglist','testlist']:
 
 781  ##        o=[]
 
 782  ##        for i in t[1::2]:
 
 783  ##            o.append(ast2string(simplify(i)))
 
 784  ##        return ','.join(o)
 
 785  ##    if t[0]=='atom':
 
 786  ####        if t[1][0]=='LPAR' and t[-1][0]=='RPAR':
 
 787  ####            return ast2string(simplify(t[2:-1]))
 
 788  ####        else:
 
 789  ##        return ensureparen(ast2string(simplify(t[2:-1])))
 
 790  ##    if t[1][0]=='trailer': # t=[[NAME,f],[trailer,[(],[ll],[)]]]
 
 791  ##        # just simplify arguments to functions
 
 792  ##        return ast2string([['NAME',t[0][1]], ['trailer',['LPAR','('],
 
 793  ##                                              simplify(t[1][2]),['RPAR',')']]])
 
 794  ##    return s
 
 795  
 
 796  
 


797 -def simplify(t):


798      return t

 799  ##    """Attempt to simplify symbolic expression string.
 
 800  ##    Adapted by R. Clewley from original DiffStr() code by Pearu Peterson and Ryan Gutenkunst.
 
 801  ##
 
 802  ##    Essentially the same format as DiffStr() except we just move down the
 
 803  ##    syntax tree calling appropriate 'do' functions on the parts to
 
 804  ##    simplify them."""
 
 805  ##    if isinstance(t, list) and len(t) == 1:
 
 806  ##        return simplify(t[0])
 
 807  ##    if t[0]=='NUMBER':
 
 808  ##        if 'e' in t[1]:
 
 809  ##            epos=t[1].find('e')
 
 810  ##            man=t[1][:epos]
 
 811  ##            ex=t[1][epos:]
 
 812  ##        else:
 
 813  ##            man=t[1]
 
 814  ##            ex=''
 
 815  ##        if man[-1] == '.':
 
 816  ##            return ['NUMBER', man+'0'+ex]
 
 817  ##        else:
 
 818  ##            return t
 
 819  ##    if t[0]=='NAME':
 
 820  ##        return t
 
 821  ##    if t[0]=='factor':
 
 822  ##        return string2ast(doneg(ensureparen(ast2string(simplify(t[2:][0])))))
 
 823  ##    if t[0]=='arith_expr':
 
 824  ##        return collect_numbers(t)
 
 825  ##    if t[0]=='term':
 
 826  ##        return collect_numbers(ensureparen_div(t))
 
 827  ##    if t[0]=='xor_expr' and t[2]=='CIRCUMFLEX': # covers alternative power syntax
 
 828  ##        alt = copy(t)
 
 829  ##        alt[0] = 'power'; alt[2]=='DOUBLESTAR'
 
 830  ##        return toCircumflexSyntax(simplify(alt))
 
 831  ##    if t[0]=='power': # covers math functions like sin, cos and log10
 
 832  ##        if t[2][0]=='trailer':
 
 833  ##            if len(t)>3:
 
 834  ##                term1 = simplify(t[:3])
 
 835  ##                if term1[0] in ['NUMBER', 'NAME']: # 'power'
 
 836  ##                    formatstr='%s'
 
 837  ##                else:
 
 838  ##                    formatstr='(%s)'
 
 839  ##                return [t[0],string2ast(formatstr%ast2string(term1)),simplify(t[3:])]
 
 840  ##            elif len(t)==3 and t[1][1]=='pow':
 
 841  ##                # 'pow' syntax case
 
 842  ##                terms = t[2][2][1::2]
 
 843  ##                return string2ast(dopower(ast2string(simplify(terms[0])),
 
 844  ##                                          ast2string(simplify(terms[1]))))
 
 845  ##            # else ignore and carry on
 
 846  ##        ts=[];o=[];
 
 847  ##        for i in t[1:]:
 
 848  ##            if i[0]=='DOUBLESTAR':
 
 849  ##                if len(o)==1: o=o[0]
 
 850  ##                ts.append(o);
 
 851  ##                o=[]
 
 852  ##            else: o.append(simplify(i))
 
 853  ##        if len(o)==1: o=o[0]
 
 854  ##        ts.append(o)
 
 855  ##        if t[2][0]=='DOUBLESTAR':
 
 856  ##            st,lft,rt=map(ast2string,[t,ts[0],ts[1]])
 
 857  ##            return string2ast(dopower(simplify_str(lft),simplify_str(rt)))
 
 858  ##        if t[2][0]=='trailer':
 
 859  ##            return simplify(ts[0])
 
 860  ##    if t[0] in ['arglist','testlist']:
 
 861  ##        o=[]
 
 862  ##        for i in t[1::2]:
 
 863  ##            o.append(ast2string(simplify(i)))
 
 864  ##        return string2ast(','.join(o))
 
 865  ##    if t[0]=='atom':
 
 866  ####        if t[1][0]=='LPAR' and t[-1][0]=='RPAR':
 
 867  ####            return simplify(t[2:-1])
 
 868  ####        else:
 
 869  ##        return string2ast(ensureparen(ast2string(simplify(t[2:-1]))))
 
 870  ##    if t[1][0]=='trailer': # t=[[NAME,f],[trailer,[(],[ll],[)]]]
 
 871  ##        # just simplify arguments to functions
 
 872  ##        return [['NAME',t[0][1]], ['trailer',['LPAR','('],
 
 873  ##                                   simplify(t[1][2]),['RPAR',')']]]
 
 874  ##    return t
 
 875  
 
 876  
 
 877  
 
 878  # ----------------------------------------------------------------------------
 
 879  
 


880 -class symbolMapClass(object):


881      """Abstract class for hassle-free symbol re-mappings.""" 


882 -    def __init__(self, symbolMap=None):


883          if isinstance(symbolMap, symbolMapClass): 
 884              self.lookupDict = copy(symbolMap.lookupDict) 
 885          elif symbolMap is None: 
 886              self.lookupDict = {} 
 887          else: 
 888              self.lookupDict = copy(symbolMap)

 889  
 


890 -    def __call__(self, arg):


891          if isinstance(arg, str): 
 892              if arg in self.lookupDict: 
 893                  return self.lookupDict[arg] 
 894              else: 
 895                  try: 
 896                      po = parserObject(arg, False) 
 897                  except: 
 898                      # cannot do anything to it!
 
 899                      return arg 
 900                  else: 
 901                      if len(po.tokenized) <= 1: 
 902                          # don't recurse, we have a single token or whitespace/CR/LF
 
 903                          return arg 
 904                      else: 
 905                          return "".join(mapNames(self,po.tokenized)) 
 906          elif hasattr(arg, 'mapNames'): 
 907              # Quantity or QuantSpec
 
 908              res = copy(arg) 
 909              res.mapNames(self) 
 910              return res 
 911          elif hasattr(arg, 'coordnames'): 
 912              # treat as point or pointset -- just transform the coordnames
 
 913              # ensure return type is the same
 
 914              res = copy(arg) 
 915              try: 
 916                  res.mapNames(self) 
 917              except AttributeError: 
 918                  raise TypeError("symbolMapClass does not know how to "
 
 919                                  "process this type of argument") 
 920              return res 
 921          elif hasattr(arg, 'iteritems'): 
 922              # ensure return type is the same
 
 923              try: 
 924                  res = copy(arg) 
 925              except TypeError: 
 926                  # not copyable, so no need to worry
 
 927                  res = arg 
 928              try: 
 929                  for k, v in arg.iteritems(): 
 930                      new_k = self.__call__(k) 
 931                      new_v = self.__call__(v) 
 932                      res[new_k] = new_v 
 933                      # delete unprocessed entry in res, from copy of arg
 
 934                      if k != new_k: 
 935                          del res[k] 
 936              except TypeError: 
 937                  # probably not a mutable type - we know what to do with a tuple
 
 938                  if isinstance(arg, tuple): 
 939                      return tuple([self.__getitem__(v) for v in arg]) 
 940                  else: 
 941                      return arg 
 942              except: 
 943                  raise TypeError("symbolMapClass does not know how to "
 
 944                                  "process this type of argument") 
 945              return res 
 946          else: 
 947              # assume arg is iterable and mutable (list, array, etc.)
 
 948              # ensure return type is the same
 
 949              try: 
 950                  res = copy(arg) 
 951              except TypeError: 
 952                  # not copyable, so no need to worry
 
 953                  res = arg 
 954              try: 
 955                  for i, v in enumerate(arg): 
 956                      # overwrite unprocessed entry in res, from copy of arg
 
 957                      res[i] = self(v) 
 958              except TypeError: 
 959                  # probably not a mutable type - we know what to do with a tuple
 
 960                  if isinstance(arg, tuple): 
 961                      return tuple([self(v) for v in arg]) 
 962                  else: 
 963                      try: 
 964                          return self.__getitem__(res) 
 965                      except: 
 966                          raise TypeError("symbolMapClass does not know how to "
 
 967                           "process this type of argument (%s)"%str(type(arg))) 
 968              except: 
 969                  try: 
 970                      return self.__getitem__(res) 
 971                  except: 
 972                      raise TypeError("symbolMapClass does not know how to "
 
 973                           "process this type of argument (%s)"%str(type(arg))) 
 974              else: 
 975                  return res

 976  
 


977 -    def __eq__(self, other):


978          try: 
 979              return self.lookupDict == other.lookupDict 
 980          except AttributeError: 
 981              return False

 982  
 


983 -    def __neq__(self, other):


984          return not self.__eq__(other)

 985  
 


986 -    def __copy__(self):


987          return symbolMapClass(deepcopy(self.lookupDict))

 988  
 


989 -    def __setitem__(self, symbol, mappedsymbol):


990          self.lookupDict[symbol] = mappedsymbol

 991  
 


992 -    def __getitem__(self, symbol):


993          try: 
 994              return self.lookupDict[symbol] 
 995          except (KeyError, TypeError): 
 996              return symbol

 997  
 


998 -    def __delitem__(self, symbol):


999          del self.lookupDict[symbol]

1000  
 


1001 -    def __contains__(self, symbol):


1002          return self.lookupDict.__contains__(symbol)

1003  
 


1004 -    def keys(self):


1005          return self.lookupDict.keys()

1006  
 


1007 -    def values(self):


1008          return self.lookupDict.values()

1009  
 


1010 -    def items(self):


1011          return self.lookupDict.items()

1012  
 


1013 -    def iterkeys(self):


1014          return self.lookupDict.iterkeys()

1015  
 


1016 -    def itervalues(self):


1017          return self.lookupDict.itervalues()

1018  
 


1019 -    def iteritems(self):


1020          return self.lookupDict.iteritems()

1021  
 


1022 -    def inverse(self):


1023          return symbolMapClass(dict(map(lambda (k,v): (v,k),
 
1024                                  self.lookupDict.iteritems())))

1025  
 


1026 -    def update(self, amap):


1027          try: 
1028              # perhaps passed a symbolMapClass object
 
1029              self.lookupDict.update(amap.lookupDict) 
1030          except AttributeError: 
1031              # was passed a dict
 
1032              self.lookupDict.update(amap)

1033  
 


1034 -    def __len__(self):


1035          return len(self.lookupDict)

1036  
 


1037 -    def copy(self):


1038          return symbolMapClass(self)

1039  
 


1040 -    def __repr__(self):


1041          return "Symbol mapping"

1042  
 
1043      __str__ = __repr__ 
1044  
 


1045 -    def has_key(self, k):


1046          return k in self.lookupDict

1047  
 
1048  
 


1049 -class auxfnDBclass(object):


1050      """Auxiliary function database, for use by parsers.""" 


1051 -    def __init__(self):


1052          self.auxnames = {}

1053  
 


1054 -    def addAuxFn(self, auxfnName, parserObj):


1055          if parserObj not in self.auxnames: 
1056              self.auxnames[parserObj] = auxfnName 
1057          else: 
1058              raise ValueError("Parser object " + parserObj.name + " already "
 
1059                               "exists in auxiliary function database")

1060  
 


1061 -    def __repr__(self):


1062          return "ModelSpec internal helper class: auxfnDBclass object"

1063  
 
1064      __str__ = __repr__ 
1065  
 


1066 -    def __call__(self, parserObj=None):


1067          if parserObj is None: 
1068              # return all auxiliary functions known
 
1069              return self.auxnames.values() 
1070          else: 
1071              try: 
1072                  return [self.auxnames[parserObj]] 
1073              except KeyError: 
1074                  return []

1075  
 


1076 -    def removeAuxFn(self, auxfnName):


1077          flagdelete = None 
1078          for k, v in self.auxnames.iteritems(): 
1079              if v == auxfnName: 
1080                  flagdelete = k 
1081                  break 
1082          if flagdelete is not None: 
1083              del self.auxnames[k]

1084  
 


1085 -    def clear(self, parserObj):


1086          if parserObj in self.auxnames: 
1087              del self.auxnames[parserObj]

1088  
 


1089 -    def clearall(self):


1090          self.auxnames = {}

1091  
 
1092  
 
1093  # only need one of these per session
 
1094  global protected_auxnamesDB 
1095  protected_auxnamesDB = auxfnDBclass() 
1096  
 
1097  
 


1098 -class parserObject(object):


1099      """Alphanumeric symbol (pseudo-)parser for mathematical expressions.
 
1100  
 
1101      An AST is not properly implemented -- rather, we tokenize,
 
1102      identify free symbols, and apply a small number of syntactic rule checks.
 
1103      The target language parser is relied upon for full syntax checking.
 
1104      """ 
1105  
 


1106 -    def __init__(self, specStr, includeProtected=True,
 
1107                   treatMultiRefs=False, ignoreTokens=[],
 
1108                   preserveSpace=False):


1109          # freeSymbols does not include protected names, and so forth.
 
1110          # freeSymbols only contains unrecognized alphanumeric symbols.
 
1111          self.usedSymbols = [] 
1112          self.freeSymbols = [] 
1113          self.preserveSpace = preserveSpace 
1114          # token by token list of the specStr
 
1115          self.tokenized = [] 
1116          if type(specStr) is str: 
1117              self.specStr = specStr 
1118          else: 
1119              print "Found type", type(specStr), ": ", specStr 
1120              raise TypeError("specStr must be a string") 
1121          self.treatMultiRefs = treatMultiRefs 
1122          # record init options in case want to reset
 
1123          self.ignoreTokens = copy(ignoreTokens) 
1124          self.includeProtected = includeProtected 
1125          # process specStr with empty symbol map to create
 
1126          # self.freeSymbols and self.usedSymbols
 
1127          self.parse(ignoreTokens, None, includeProtected, reset=True)

1128  
 
1129  
 


1130 -    def isCompound(self, ops=['+', '-', '*', '/']):


1131          """Function to verify whether an expression is 'compound',
 
1132          in the sense that it has an operator at the root of its syntax
 
1133          parse tree (i.e. not inside braces).""" 
1134          result = False 
1135          nested = 0 
1136          if len(self.tokenized) > 2: 
1137              stage = 0 
1138              for s in self.tokenized: 
1139                  if stage == 0: 
1140                      if s in self.usedSymbols and nested == 0: 
1141                          stage = 1 
1142                      elif s == ')': 
1143                          stage = 1 
1144                          nested = max([0, nested-1]) 
1145                      elif s == '(': 
1146                          nested += 1 
1147                  elif stage == 1: 
1148                      if s in ops and nested == 0: 
1149                          stage = 2 
1150                      elif s == '(': 
1151                          nested += 1 
1152                      elif s == ')': 
1153                          nested = max([0, nested-1]) 
1154                      elif nested == 0: 
1155                          stage = 0 
1156                  elif stage == 2: 
1157                      if s in self.usedSymbols and nested == 0: 
1158                          stage = 3 
1159                      elif s == '(': 
1160                          stage = 3 
1161                          nested += 1 
1162                      elif s == ')': 
1163                          nested = max([0, nested-1]) 
1164                      elif nested == 0: 
1165                          stage = 0 
1166                  if stage == 3: 
1167                      result = True 
1168                      break 
1169          return result

1170  
 


1171 -    def __call__(self, specialtoks=None, symbolMap=None, includeProtected=True):


1172          if specialtoks is None: 
1173              if self.ignoreTokens is not None: 
1174                  specialtoks = self.ignoreTokens 
1175              else: 
1176                  specialtoks = [] 
1177          if self.tokenized == []: 
1178              return self.parse(specialtoks, symbolMap, includeProtected) 
1179          else: 
1180              if symbolMap is None: 
1181                  return "".join(self.tokenized) 
1182              else: 
1183                  return "".join(symbolMap(self.tokenized))

1184  
 


1185 -    def find(self, token):


1186          """Find all occurrences of the given token in the expression, returning a list
 
1187          of indices (empty if not present).
 
1188          """ 
1189          if self.tokenized == []: 
1190              self.parse([]) 
1191          return [i for i, t in enumerate(self.tokenized) if t == token]

1192  
 


1193 -    def parse(self, specialtoks, symbolMap=None, includeProtected=True,
 
1194                reset=False):


1195          if reset: 
1196              self.usedSymbols = [] 
1197              self.freeSymbols = [] 
1198          if symbolMap is None: 
1199              # dummy identity function
 
1200              symbolMap = lambda x: x 
1201          specialtokens = specialtoks + ['('] + self.usedSymbols 
1202          if includeProtected: 
1203              specialtokens.extend(protected_allnames) 
1204              protected_auxnames = protected_auxnamesDB(self) 
1205          else: 
1206              protected_auxnames = [] 
1207          if self.treatMultiRefs: 
1208              specialtokens.append('[') 
1209          dohierarchical = '.' not in specialtokens 
1210          allnames = specialtokens + protected_auxnames 
1211          specstr = self.specStr   # eases notation 
1212          returnstr = "" 
1213          if specstr == "": 
1214              # Hack for empty specs to pass without losing their last characters
 
1215              specstr = " " 
1216          elif specstr[-1] != ')': 
1217              # temporary hack because strings not ending in ) lose their last
 
1218              # character!
 
1219              # Problem could be with line "if scount < speclen - 1:" below
 
1220              # ... should it be <= ?
 
1221              specstr += " " 
1222          scount = 0 
1223          speclen = len(specstr) 
1224          # temp holders for used and free symbols
 
1225          used = copy(self.usedSymbols) 
1226          free = copy(self.freeSymbols) 
1227          tokenized = [] 
1228          # current token being built is: s
 
1229          # current character being processed is: stemp
 
1230          s = '' 
1231          foundtoken = False 
1232          while scount < speclen: 
1233              stemp = specstr[scount] 
1234              ## DEBUGGING PRINT STATEMENTS
 
1235  ##            print "\n*********************************************"
 
1236  ##            print specstr[:scount]
 
1237  ##            print stemp
 
1238  ##            print s
 
1239  ##            print tokenized
 
1240              scount += 1 
1241              if name_chars_RE.match(stemp) is None: 
1242                  # then stemp is a non-alphanumeric char
 
1243                  if s not in ['', ' ', '\n', '\t']: 
1244                      # checking allnames catches var names etc. that are valid
 
1245                      # in auxiliary functions but are not special tokens
 
1246                      # and must be left alone
 
1247                      if s in allnames: 
1248                          snew = symbolMap(s) 
1249                          tokenized.append(snew) 
1250                          if snew not in used: 
1251                              used.append(snew) 
1252                          returnstr += snew 
1253                      else: 
1254                          # isdecimal cases:
 
1255                          #  s = number followed by a '.'
 
1256                          #  s = number with 'e' followed by +/-
 
1257                          #      (just a number dealt with elsewhere)
 
1258                          isnumtok = isNumericToken(s) 
1259                          issimpledec = stemp == '.' and isnumtok 
1260                          isexpdec = s[-1] in ['e','E'] and s[0] not in ['e','E']\
 
1261                               and stemp in ['-','+'] and isnumtok 
1262                          isdecimal = issimpledec or isexpdec 
1263                          ishierarchicalname = stemp == '.' and isNameToken(s) 
1264                          if isdecimal or (ishierarchicalname and dohierarchical): 
1265                              # continue building token
 
1266                              s += stemp 
1267                              continue 
1268                          else: 
1269                              # We have found a complete token
 
1270                              snew = symbolMap(s) 
1271                              if s[0] not in num_chars + ['+','-'] \
 
1272                                      and snew not in free: 
1273                                  free.append(snew) 
1274                              tokenized.append(snew) 
1275                              if snew not in used: 
1276                                  used.append(snew) 
1277                              returnstr += snew 
1278                  if stemp in ['+', '-']: 
1279                      # may be start of a unary number
 
1280                      try: 
1281                          next_stemp = specstr[scount] 
1282                      except IndexError: 
1283                          tokenized.append(stemp) 
1284                          returnstr += stemp 
1285                          s = '' 
1286                          continue 
1287                      if (tokenized==[] or tokenized[-1]=='(') and \
 
1288                         next_stemp in num_chars + ['.']: 
1289                          # continue to build token
 
1290                          s += stemp 
1291                          continue 
1292                      elif len(tokenized)>0 and tokenized[-1] == '+': 
1293                          # process double sign
 
1294                          if stemp == '-': 
1295                              tokenized[-1] = '-' 
1296                              returnstr = returnstr[:-1] + '-' 
1297                          # else do nothing -- keep just one '+'
 
1298                          s = '' 
1299                          continue 
1300                      elif len(tokenized)>0 and tokenized[-1] == '-': 
1301                          # process double sign
 
1302                          if stemp == '-': 
1303                              tokenized[-1] = '+' 
1304                              returnstr = returnstr[:-1] + '+' 
1305                          # else do nothing -- keep just one '-'
 
1306                          s = '' 
1307                          continue 
1308                      else: 
1309                          tokenized.append(stemp) 
1310                          returnstr += stemp 
1311                          s = '' 
1312                          continue 
1313                  elif stemp in ['`', '!', '@', '#', '$', '{',
 
1314                                 '}', "\\"]: 
1315                      if stemp in specialtokens: 
1316                          tokenized.append(stemp) 
1317                          returnstr += stemp 
1318                          s = '' 
1319                          continue 
1320                      else: 
1321  ##                        if stemp == '^':
 
1322  ##                            raise ValueError('Symbol ^ is not allowed. '
 
1323  ##                                             'Please use the pow() call')
 
1324  ##                        else:
 
1325                          print "Problem with string '%s'"%specstr 
1326                          raise ValueError('Symbol %s is illegal. '%stemp) 
1327                  elif stemp == '[': 
1328                      # self.treatMultiRefs == False and '[' in specialtokens
 
1329                      # means it was probably in the ignoreToken list in __init__
 
1330                      if self.treatMultiRefs and len(tokenized)>0 \
 
1331                              and (tokenized[-1].isalnum() or \
 
1332                                   ('[' in specialtokens and not ( \
 
1333                                    isVectorClause(specstr[scount-1:]) or \
 
1334                                    len(tokenized)>1 and tokenized[-2] in \
 
1335                                    ('max', 'min', 'max_', 'min_')))): 
1336                          # then this is probably an actual multiRef
 
1337                          s = '[' 
1338                      elif '[' in specialtokens: 
1339                          returnstr += '[' 
1340                          # s already to tokenized in this case
 
1341                          tokenized.append('[')   # was just '[' 
1342                          s = '' 
1343                          continue 
1344                      else: 
1345                          raise ValueError("Syntax error: Square braces not to "
 
1346                                           "be used outside of multiple Quantity"
 
1347                                           " definitions and references") 
1348                  # only use next clause if want to process function call
 
1349                  # arguments specially
 
1350  #                elif stemp == '(':
 
1351  #                    returnstr += s
 
1352  #                    s = stemp
 
1353                  else: 
1354                      if stemp == "*": 
1355                          if len(returnstr)>1 and returnstr[-1] == "*": 
1356                              # check for ** case
 
1357                              if tokenized[-1] == '*': 
1358                                  tokenized[-1] = '**' 
1359                              else: 
1360                                  tokenized.append('**') 
1361                              s = '' 
1362                              returnstr += stemp 
1363                              continue   # avoids returnstr += stemp below 
1364  ##                            if "**" in specialtokens:
 
1365  ##                                if tokenized[-1] == '*':
 
1366  ##                                    tokenized[-1] = '**'
 
1367  ##                                else:
 
1368  ##                                    tokenized.append('**')
 
1369  ##                                s = ''
 
1370  ##                                returnstr += "**"
 
1371  ##                                continue   # avoids returnstr += stemp below
 
1372  ##                            else:
 
1373  ##                                raise ValueError('Operator ** is not allowed. '
 
1374  ##                                       'Please use the pow() call')
 
1375                          else: 
1376                              # just a single *
 
1377                              tokenized.append('*') 
1378                      elif stemp == "=": 
1379                          if len(returnstr)>1: 
1380                               # check for >= and <= cases
 
1381                              if returnstr[-1] == ">": 
1382                                  if tokenized[-1] == '>': 
1383                                      tokenized[-1] = '>=' 
1384                                  else: 
1385                                      tokenized.append('>=') 
1386                                  s = '' 
1387                                  returnstr += stemp 
1388                                  continue   # avoids returnstr += stemp below 
1389                              elif returnstr[-1] == "<": 
1390                                  if tokenized[-1] == '<': 
1391                                      tokenized[-1] = '<=' 
1392                                  else: 
1393                                      tokenized[-1] = '<=' 
1394                                  s = '' 
1395                                  returnstr += stemp 
1396                                  continue   # avoids returnstr += stemp below 
1397                              else: 
1398                                  tokenized.append('=') 
1399                          else: 
1400                              tokenized.append('=') 
1401                      elif stemp in [" ","\t","\n"]: 
1402                          if self.preserveSpace: tokenized.append(stemp) 
1403                      else: 
1404                          tokenized.append(stemp) 
1405                      s = '' 
1406                      returnstr += stemp 
1407                      continue 
1408              else: 
1409                  s += stemp 
1410              if s in specialtokens: 
1411                  # only use next clause if want to process function call
 
1412                  # arguments specially
 
1413  #                if s == '(':
 
1414  #                    foundtoken = False  # so that don't enter next if statement
 
1415  #                    tokenized.append(s)
 
1416  #                    returnstr += s
 
1417  #                    s = ''
 
1418                  if s == '[' and self.treatMultiRefs and len(tokenized)>0 \
 
1419                          and (tokenized[-1].isalnum() or \
 
1420                               ('[' in specialtokens and not isVectorClause(specstr[scount-1:]))): 
1421                      # then this is probably an actual multiRef ...
 
1422                      # will treat as multiRef if there's an alphanumeric
 
1423                      # token directly preceding '[', e.g. z[i,0,1]
 
1424                      # or if commas are not inside,
 
1425                      # otherwise would catch vector usage, e.g. [[x,y],[a,b]]
 
1426                      foundtoken = False    # don't go into next clause 
1427                      # copy the square-bracketed clause to the output
 
1428                      # verbatim, and add whole thing as a token.
 
1429                      try: 
1430                          rbpos = specstr[scount:].index(']') 
1431                      except ValueError: 
1432                          raise ValueError("Mismatch [ and ] in spec") 
1433                      # expr includes both brackets
 
1434                      expr = specstr[scount-1:scount+rbpos+1] 
1435                      # find index name in this expression. this should
 
1436                      # be the only free symbol, otherwise syntax error.
 
1437                      temp = parserObject(expr[1:-1],
 
1438                                          includeProtected=False) 
1439                      if len(temp.freeSymbols) == 1: 
1440                          free.extend(temp.freeSymbols) 
1441                          # not sure whether should add to usedSymbols
 
1442                          # for consistency with freeSymbols, or leave
 
1443                          # it out for consistency with tokenized
 
1444  #                                used.append(temp.freeSymbols)
 
1445                      else: 
1446                          raise ValueError("Invalid index clause in "
 
1447                                           "multiple quantity reference -- "
 
1448                                           "multiple index names used in [...]") 
1449                      # start next parsing iteration after the ']'
 
1450                      scount += rbpos+1 
1451                      # treat whole expression as one symbol
 
1452                      returnstr += expr 
1453                      tokenized.append(expr) 
1454                      used.append(expr) 
1455                      s = '' 
1456                  else: 
1457                      if scount < speclen - 1: 
1458                          if name_chars_RE.match(specstr[scount]) is None: 
1459                              foundtoken = True 
1460                          else: 
1461                              if s[-1] in ['e','E'] and s[0] not in ['e','E'] and \
 
1462                                 name_chars_RE.match(specstr[scount]).group() \
 
1463                                         in num_chars+['-','+']: 
1464                                  # not expecting an arithmetic symbol or space
 
1465                                  # ... we *are* expecting a numeric
 
1466                                  foundtoken = True 
1467                      else: 
1468                          foundtoken = True 
1469                  if foundtoken: 
1470                      if includeProtected: 
1471                          if s == 'for': 
1472                              # check next char is '('
 
1473                              if specstr[scount] != '(': 
1474                                  print "Next char found:", specstr[scount] 
1475                                  raise ValueError("Invalid 'for' macro syntax") 
1476                              # find next ')' (for statement should contain no
 
1477                              # braces itself)
 
1478                              try: 
1479                                  rbpos = specstr[scount:].index(')') 
1480                              except ValueError: 
1481                                  raise ValueError("Mismatch ( and ) in 'for' "
 
1482                                                   "macro") 
1483                              # expr includes both brackets
 
1484                              expr = specstr[scount:scount+rbpos+1] 
1485                              # find index name in this expression. this should
 
1486                              # be the only free symbol, otherwise syntax error.
 
1487                              temp = parserObject(expr, includeProtected=False) 
1488                              macrotests = [len(temp.tokenized) == 7,
 
1489                                 temp.tokenized[2] == temp.tokenized[4] == ',',
 
1490                                 temp.tokenized[5] in ['+','*']] 
1491                              if not alltrue(macrotests): 
1492                                  print "specstr was: ", specstr 
1493                                  print "tokens: ", temp.tokenized 
1494                                  print "test results: ", macrotests 
1495                                  raise ValueError("Invalid sub-clause in "
 
1496                                                   "'for' macro") 
1497                              # start next parsing iteration after the ')'
 
1498                              scount += rbpos+1 
1499                              # keep contents of braces as one symbol
 
1500                              returnstr += s+expr 
1501                              tokenized.extend([s,expr]) 
1502                              if s not in used: 
1503                                  used.append(s) 
1504                              if expr not in used: 
1505                                  used.append(expr) 
1506                          elif s == 'abs': 
1507                              snew = symbolMap(s) 
1508                              returnstr += snew 
1509                              tokenized.append(snew) 
1510                              if snew not in used: 
1511                                  used.append(snew) 
1512                          elif s in protected_scipynames + protected_specialfns: 
1513                              snew = symbolMap(s) 
1514                              returnstr += snew 
1515                              tokenized.append(snew) 
1516                              if snew not in used: 
1517                                  used.append(snew) 
1518                          elif s in protected_mathnames: 
1519                              if s in ['e','E']: 
1520                                  # special case where e is either = exp(0)
 
1521                                  # as a constant or it's an exponent in 1.0e-4
 
1522                                  if len(returnstr)>0: 
1523                                      if returnstr[-1] not in num_chars+['.']: 
1524                                          snew = symbolMap(s.lower()) 
1525                                          returnstr += snew 
1526                                          tokenized.append(snew) 
1527                                          if snew not in used: 
1528                                              used.append(snew) 
1529                                      else: 
1530                                          returnstr += s 
1531                                  else: 
1532                                      snew = symbolMap(s.lower()) 
1533                                      returnstr += snew 
1534                                      tokenized.append(snew) 
1535                                      if snew not in used: 
1536                                          used.append(snew) 
1537                              else: 
1538                                  snew = symbolMap(s) 
1539                                  returnstr += snew 
1540                                  tokenized.append(snew) 
1541                                  if snew not in used: 
1542                                      used.append(snew) 
1543                          elif s in protected_randomnames: 
1544                              snew = symbolMap(s) 
1545                              returnstr += snew 
1546                              tokenized.append(snew) 
1547                              if snew not in used: 
1548                                  used.append(snew) 
1549                          elif s in protected_auxnames: 
1550                              snew = symbolMap(s) 
1551                              returnstr += snew 
1552                              tokenized.append(snew) 
1553                          else: 
1554                              # s is e.g. a declared argument to an aux fn but
 
1555                              # only want to ensure it is present. no action to
 
1556                              # take.
 
1557                              snew = symbolMap(s) 
1558                              tokenized.append(snew) 
1559                              returnstr += snew 
1560                      else: 
1561                          # not includeProtected names case, so just map
 
1562                          # symbol
 
1563                          snew = symbolMap(s) 
1564                          tokenized.append(snew) 
1565                          returnstr += snew 
1566                      # reset for next iteration
 
1567                      s = '' 
1568                      foundtoken = False 
1569          # end of scount while loop
 
1570          if reset: 
1571              # hack to remove any string literals
 
1572              actual_free = [sym for sym in free if sym in tokenized] 
1573              for sym in [sym for sym in free if sym not in actual_free]: 
1574                  is_literal = False 
1575                  for tok in tokenized: 
1576                      # does symbol appear in quotes inside token?
 
1577                      # if so, then it's a literal and not a free symbol
 
1578                      if sym in tok: 
1579                          start_ix = tok.index(sym) 
1580                          end_ix = start_ix + len(sym) - 1 
1581                          if len(tok) > end_ix and start_ix > 0: 
1582                              # then symbol is embedded inside the string
 
1583                              doub = tok[start_ix-1] == tok[end_ix+1] == '"' 
1584                              sing = tok[start_ix-1] == tok[end_ix+1] == "'" 
1585                              is_literal = doub or sing 
1586                  if not is_literal: 
1587                      actual_free.append(sym) 
1588              self.usedSymbols = used 
1589              self.freeSymbols = actual_free 
1590              self.specStr = returnstr 
1591              self.tokenized = tokenized 
1592          # strip extraneous whitespace
 
1593          return returnstr.strip()

1594  
 
1595  
 
1596  #-----------------------------------------------------------------------------
 
1597  
 
1598  
 


1599 -def isToken(s, treatMultiRefs=False):


1600      # token must be alphanumeric string (with no punctuation, operators, etc.)
 
1601      if not isinstance(s, str): 
1602          return False 
1603      try: 
1604          temp = parserObject(s, includeProtected=False,
 
1605                              treatMultiRefs=treatMultiRefs) 
1606      except ValueError: 
1607          return False 
1608      if treatMultiRefs and s.find('[')>0: 
1609          lenval = 2 
1610      else: 
1611          lenval = 1 
1612      return not temp.isCompound() and len(temp.usedSymbols) == lenval \
 
1613              and len(temp.freeSymbols) == lenval \
 
1614              and len(temp.tokenized) == lenval

1615  
 
1616  
 


1617 -def isNameToken(s, treatMultiRefs=False):


1618      return isToken(s, treatMultiRefs=treatMultiRefs) \
 
1619          and s[0] not in num_chars and not (len(s)==1 and s=="_")

1620  
 


1621 -def isIntegerToken(arg):


1622      return alltrue([t in '0123456789' for t in arg])

1623  
 


1624 -def isNumericToken(arg):


1625      # supports unary + / - at front, and checks for usage of exponentials
 
1626      # (using 'E' or 'e')
 
1627      try: 
1628          s = arg.lower() 
1629      except AttributeError: 
1630          return False 
1631      try: 
1632          if s[0] in ['+','-']: 
1633              s_rest = s[1:] 
1634          else: 
1635              s_rest = s 
1636      except IndexError: 
1637          return False 
1638      pts = s.count('.') 
1639      exps = s.count('e') 
1640      pm = s_rest.count('+') + s_rest.count('-') 
1641      if pts > 1 or exps > 1 or pm > 1: 
1642          return False 
1643      if exps == 1: 
1644          exp_pos = s.find('e') 
1645          pre_exp = s[:exp_pos] 
1646          # must be numbers before and after the 'e'
 
1647          if not sometrue([n in num_chars for n in pre_exp]): 
1648              return False 
1649          if s[-1]=='e': 
1650              # no chars after 'e'!
 
1651              return False 
1652          if not sometrue([n in num_chars for n in s[exp_pos:]]): 
1653              return False 
1654          # check that any additional +/- occurs directly after 'e'
 
1655          if pm == 1: 
1656              pm_pos = max([s_rest.find('+'), s_rest.find('-')]) 
1657              if s_rest[pm_pos-1] != 'e': 
1658                  return False 
1659              e_rest = s_rest[pm_pos+1:]   # safe due to previous check 
1660          else: 
1661              e_rest = s[exp_pos+1:] 
1662          # only remaining chars in s after e and possible +/- are numbers
 
1663          if '.' in e_rest: 
1664              return False 
1665      # cannot use additional +/- if not using exponent
 
1666      if pm == 1 and exps == 0: 
1667          return False 
1668      return alltrue([n in num_chars + ['.', 'e', '+', '-'] for n in s_rest])

1669  
 
1670  
 


1671 -def findNumTailPos(s):


1672      """Find position of numeric tail in alphanumeric string.
 
1673  
 
1674      e.g. findNumTailPos('abc678') = 3""" 
1675      try: 
1676          l = len(s) 
1677          if l > 1: 
1678              if s[-1] not in num_chars or s[0] in num_chars: 
1679                  raise ValueError("Argument must be an alphanumeric string "
 
1680                                "starting with a letter and ending in a number") 
1681              for i in range(1, l+1): 
1682                  if s[-i] not in num_chars: 
1683                      return l-i+1 
1684          else: 
1685              raise ValueError("Argument must be alphanumeric string starting "
 
1686                               "with a letter and ending in a number") 
1687      except TypeError: 
1688          raise ValueError("Argument must be alphanumeric string starting "
 
1689                               "with a letter and ending in a number")

1690  
 
1691  
 


1692 -def isHierarchicalName(s, sep=NAMESEP, treatMultiRefs=False):


1693      s_split = s.split(sep) 
1694      return len(s_split) > 1 and alltrue([isNameToken(t, treatMultiRefs) \
 
1695                                           for t in s_split])

1696  
 


1697 -def isVectorClause(s):


1698      brace = findEndBrace(s, '[',']') 
1699      if s[0] == '[' and isinstance(brace, int): 
1700          return ',' in s[1:brace]

1701  
 
1702  
 


1703 -def replaceSepList(speclist):


1704      return [replaceSep(spec) for spec in speclist]

1705  
 


1706 -def replaceSepListInv(speclist):


1707      return [replaceSepInv(spec) for spec in speclist]

1708  
 


1709 -def replaceSepInv(spec):


1710      """Invert default name separator replacement.""" 
1711      return replaceSep(spec, "_", NAMESEP)

1712  
 
1713  
 


1714 -def replaceSep(spec, sourcesep=NAMESEP, targetsep="_"):


1715      """Replace hierarchy separator character with another and return spec
 
1716      string. e.g. "." -> "_"
 
1717      Only replaces the character between name tokens, not between numbers.""" 
1718  
 
1719      for char in sourcesep: 
1720          if alphabet_chars_RE.match(char) is not None: 
1721              raise ValueError("Source separator must be non-alphanumeric") 
1722      for char in targetsep: 
1723          if alphabet_chars_RE.match(char) is not None: 
1724              raise ValueError("Target separator must be non-alphanumeric") 
1725      if isinstance(spec, str): 
1726          return replaceSepStr(spec, sourcesep, targetsep) 
1727      else: 
1728          # safe way to get string definition from either Variable or QuantSpec
 
1729          return replaceSepStr(str(spec()), sourcesep, targetsep)

1730  
 
1731  
 


1732 -def mapNames(themap, target):


1733      """Map names in <target> argument using the symbolMapClass
 
1734      object <themap>, returning a renamed version of the target.
 
1735      N.B. Only maps the keys of a dictionary type""" 
1736      try: 
1737          themap.lookupDict 
1738      except AttributeError: 
1739          t = repr(type(themap)) 
1740          raise TypeError("Map argument must be of type symbolMapClass, not %s"%t) 
1741      if hasattr(target, 'mapNames'): 
1742          ct = copy(target) 
1743          ct.mapNames(themap)  # these methods work in place 
1744          return ct 
1745      elif isinstance(target, list): 
1746          return themap(target) 
1747      elif isinstance(target, tuple): 
1748          return tuple(themap(target)) 
1749      elif hasattr(target, 'iteritems'): 
1750          o = {} 
1751          for k, v in target.iteritems(): 
1752              o[themap(k)] = v 
1753          return o 
1754      elif isinstance(target, str): 
1755          return themap(target) 
1756      elif target is None: 
1757          return None 
1758      else: 
1759          raise TypeError("Invalid target type %s"%repr(type(target)))

1760  
 
1761  
 
1762  ## internal functions for replaceSep
 


1763 -def replaceSepQSpec(spec, sourcesep, targetsep):


1764      # currently unused because many QuantSpec's with hierarchical names
 
1765      # are not properly tokenized! They may have the tokenized form
 
1766      # ['a_parent','.','a_child', <etc.>] rather than
 
1767      # ['a_parent.a_child', <etc.>]
 
1768      outstr = "" 
1769      # search for pattern <nameToken><sourcechar><nameToken>
 
1770      try: 
1771          for t in spec[:]: 
1772              if isHierarchicalName(t, sourcesep): 
1773                  outstr += t.replace(sourcesep, targetsep) 
1774              else: 
1775                  # just return original token
 
1776                  outstr += t 
1777      except AttributeError: 
1778          raise ValueError("Invalid QuantSpec passed to replaceSep()") 
1779      return outstr

1780  
 
1781  
 


1782 -def replaceSepStr(spec, sourcesep, targetsep):


1783      # spec is a string (e.g. 'leaf.v'), and p.tokenized contains the
 
1784      # spec split by the separator (e.g. ['leaf', '.', 'v']) if the separator
 
1785      # is not "_", otherwise it will be retained as part of the name
 
1786      outstr = "" 
1787      treatMultiRefs = '[' in spec and ']' in spec 
1788      p = parserObject(spec, treatMultiRefs=treatMultiRefs,
 
1789                       includeProtected=False) 
1790      # search for pattern <nameToken><sourcechar><nameToken>
 
1791      # state 0: not in pattern
 
1792      # state 1: found nameToken
 
1793      # state 2: found nameToken then sourcechar
 
1794      # state 3: found complete pattern
 
1795      state = 0 
1796      for t in p.tokenized: 
1797          if isNameToken(t): 
1798              if sourcesep in t: 
1799                  # in case sourcesep == '_'
 
1800                  tsplit = t.split(sourcesep) 
1801                  if alltrue([isNameToken(ts) for ts in tsplit]): 
1802                      outstr += targetsep.join(tsplit) 
1803                  else: 
1804                      outstr += t 
1805              else: 
1806                  if state == 0: 
1807                      state = 1 
1808                      outstr += t 
1809                  elif state == 1: 
1810                      state = 0 
1811                      outstr += t 
1812                  else: 
1813                      # state == 2
 
1814                      state = 1   # in case another separator follows 
1815                      outstr += targetsep + t 
1816              continue 
1817          elif t == sourcesep: 
1818              if state == 1: 
1819                  state = 2 
1820                  # delay output until checked next token
 
1821              else: 
1822                  # not part of pattern, so reset
 
1823                  state = 0 
1824                  outstr += t 
1825          else: 
1826              state = 0 
1827              outstr += t 
1828      return outstr

1829  
 
1830  
 


1831 -def joinStrs(strlist):


1832      """Join a list of strings into a single string (in order).""" 
1833  
 
1834      return ''.join(strlist)

1835  
 
1836  
 


1837 -def joinAsStrs(objlist,sep=""):


1838      """Join a list of objects in their string representational form.""" 
1839  
 
1840      retstr = '' 
1841      for o in objlist: 
1842          if type(o) is str: 
1843              retstr += o + sep 
1844          else: 
1845              retstr += str(o) + sep 
1846      avoidend = len(sep) 
1847      if avoidend > 0: 
1848          return retstr[:-avoidend] 
1849      else: 
1850          return retstr

1851  
 
1852  
 


1853 -def count_sep(specstr, sep=','):


1854      """Count number of specified separators (default = ',') in given string,
 
1855      avoiding occurrences of the separator inside nested braces""" 
1856  
 
1857      num_seps = 0 
1858      brace_depth = 0 
1859      for s in specstr: 
1860          if s == sep and brace_depth == 0: 
1861              num_seps += 1 
1862          elif s == '(': 
1863              brace_depth += 1 
1864          elif s == ')': 
1865              brace_depth -= 1 
1866      return num_seps

1867  
 
1868  
 


1869 -def parseMatrixStrToDictStr(specstr, specvars, m=0):


1870      """Convert string representation of m-by-n matrix into a single
 
1871      string, assuming a nested comma-delimited list representation in
 
1872      the input, and outputting a dictionary of the sub-lists, indexed
 
1873      by the ordered list of names specvars (specified as an
 
1874      argument).""" 
1875  
 
1876      specdict = {} 
1877      # matrix is n by m
 
1878      n = len(specvars) 
1879      if n == 0: 
1880          raise ValueError("parseMatrixStrToDictStr: specvars was empty") 
1881      if m == 0: 
1882          # assume square matrix
 
1883          m = len(specvars) 
1884      # strip leading and trailing whitespace
 
1885      spectemp1 = specstr.strip() 
1886      assert spectemp1[0] == '[' and spectemp1[-1] == ']', \
 
1887          ("Matrix must be supplied as a Python matrix, using [ and ] syntax") 
1888      # strip first [ and last ] and then all whitespace and \n
 
1889      spectemp2 = spectemp1[1:-1].replace(' ','').replace('\n','') 
1890      splitdone = False 
1891      entrycount = 0 
1892      startpos = 0 
1893      try: 
1894          while not splitdone: 
1895              nextrbrace = findEndBrace(spectemp2[startpos:],
 
1896                                         '[', ']') + startpos 
1897              if nextrbrace is None: 
1898                  raise ValueError("Mismatched braces before end of string") 
1899              specdict[specvars[entrycount]] = \
 
1900                                  spectemp2[startpos:nextrbrace+1] 
1901              entrycount += 1 
1902              if entrycount < n: 
1903                  nextcomma = spectemp2.find(',', nextrbrace) 
1904                  if nextcomma > 0: 
1905                      nextlbrace = spectemp2.find('[', nextcomma) 
1906                      if nextlbrace > 0: 
1907                          startpos = nextlbrace 
1908                      else: 
1909                          raise ValueError("Not enough comma-delimited entries") 
1910                  else: 
1911                      raise ValueError("Not enough comma-delimited entries") 
1912              else: 
1913                  splitdone = True 
1914      except: 
1915          print "Error in matrix specification" 
1916          raise 
1917      return specdict

1918  
 
1919  
 


1920 -def readArgs(argstr, lbchar='(', rbchar=')'):


1921      """Parse arguments out of string beginning and ending with braces
 
1922      (default: round brace).
 
1923  
 
1924      Returns a triple: [success_boolean, list of arguments, number of args]""" 
1925      bracetest = argstr[0] == lbchar and argstr[-1] == rbchar 
1926      rest = argstr[1:-1].replace(" ","") 
1927      pieces = [] 
1928      while True: 
1929          if '(' in rest: 
1930              lix = rest.index('(') 
1931              rix = findEndBrace(rest[lix:]) + lix 
1932              new = rest[:lix].split(",") 
1933              if len(pieces) > 0: 
1934                  pieces[-1] = pieces[-1] + new[0] 
1935                  pieces.extend(new[1:]) 
1936              else: 
1937                  pieces.extend(new) 
1938              if len(pieces) > 0: 
1939                  pieces[-1] = pieces[-1] + rest[lix:rix+1] 
1940              else: 
1941                  pieces.append(rest[lix:rix+1]) 
1942              rest = rest[rix+1:] 
1943          else: 
1944              new = rest.split(",") 
1945              if len(pieces) > 0: 
1946                  pieces[-1] = pieces[-1] + new[0] 
1947                  pieces.extend(new[1:]) 
1948              else: 
1949                  pieces.extend(new) 
1950              # quit while loop
 
1951              break 
1952      return [bracetest, pieces, len(argstr)]

1953  
 
1954  
 


1955 -def findEndBrace(s, lbchar='(', rbchar=')'):


1956      """Find position in string (or list of strings), s, at which final matching
 
1957      brace occurs (if at all). If not found, returns None.
 
1958  
 
1959      s[0] must be the left brace character. Default left and right braces are
 
1960      '(' and ')'. Change them with the optional second and third arguments.
 
1961      """ 
1962      pos = 0 
1963      assert s[0] == lbchar, 'string argument must begin with left brace' 
1964      stemp = s 
1965      leftbrace_count = 0 
1966      notDone = True 
1967      while len(stemp) > 0 and notDone: 
1968          # for compatibility with s being a list, use index method
 
1969          try: 
1970              left_pos = stemp.index(lbchar) 
1971          except ValueError: 
1972              left_pos = -1 
1973          try: 
1974              right_pos = stemp.index(rbchar) 
1975          except ValueError: 
1976              right_pos = -1 
1977          if left_pos >= 0: 
1978              if left_pos < right_pos: 
1979                  if left_pos >= 0: 
1980                      leftbrace_count += 1 
1981                      pos += left_pos+1 
1982                      stemp = s[pos:] 
1983                  else: 
1984                      # no left braces found. next brace is right.
 
1985                      if leftbrace_count > 0: 
1986                          leftbrace_count -= 1 
1987                          pos += right_pos+1 
1988                          stemp = s[pos:] 
1989              else: 
1990                  # right brace found first
 
1991                  leftbrace_count -= 1 
1992                  pos += right_pos+1 
1993                  stemp = s[pos:] 
1994          else: 
1995              if right_pos >= 0: 
1996                  # right brace found first
 
1997                  leftbrace_count -= 1 
1998                  pos += right_pos+1 
1999                  stemp = s[pos:] 
2000              else: 
2001                  # neither were found (both == -1)
 
2002                  raise ValueError('End of string found before closing brace') 
2003          if leftbrace_count == 0: 
2004              notDone = False 
2005              # adjust for
 
2006              pos -= 1 
2007      if leftbrace_count == 0: 
2008          return pos 
2009      else: 
2010          return None

2011  
 
2012  
 


2013 -def makeParList(objlist, prefix=''):


2014      """wrap objlist into a comma separated string of str(objects)""" 
2015      parlist = ', '.join(map(lambda i: prefix+str(i), objlist)) 
2016      return parlist

2017  
 
2018  
 


2019 -def wrapArgInCall(source, callfn, wrapL, wrapR=None, argnums=[0],
 
2020                    notFirst=False):


2021      """Add delimiters to single argument in function call.""" 
2022      done = False 
2023      output = "" 
2024      currpos = 0 
2025      first_occurrence = True 
2026      if wrapR is None: 
2027          # if no specific wrapR is specified, just use wrapL
 
2028          # e.g. for symmetric delimiters such as quotes
 
2029          wrapR = wrapL 
2030      assert isinstance(wrapL, str) and isinstance(wrapR, str), \
 
2031             "Supplied delimiters must be strings" 
2032      while not done: 
2033          # find callfn in source
 
2034          findposlist = [source[currpos:].find(callfn+'(')] 
2035          try: 
2036              findpos = min(filter(lambda x:x>=0,findposlist))+currpos 
2037          except ValueError: 
2038              done = True 
2039          if not done: 
2040              # find start and end braces
 
2041              startbrace = source[findpos:].find('(')+findpos 
2042              endbrace = findEndBrace(source[startbrace:])+startbrace 
2043              output += source[currpos:startbrace+1] 
2044              # if more than one argument present, apply wrapping to specified
 
2045              # arguments
 
2046              currpos = startbrace+1 
2047              numargs = source[startbrace+1:endbrace].count(',')+1 
2048              if max(argnums) >= numargs: 
2049                  raise ValueError("Specified argument number out of range") 
2050              if numargs > 1: 
2051                  for argix in range(numargs-1): 
2052                      nextcomma = source[currpos:endbrace].find(',') 
2053                      argstr = source[currpos:currpos + nextcomma] 
2054                      # get rid of leading or tailing whitespace
 
2055                      argstr = argstr.strip() 
2056                      if argix in argnums: 
2057                          if first_occurrence and notFirst: 
2058                              output += source[currpos:currpos + nextcomma + 1] 
2059                          else: 
2060                              output += wrapL + argstr + wrapR + ',' 
2061                          first_occurrence = False 
2062                      else: 
2063                          output += source[currpos:currpos + nextcomma + 1] 
2064                      currpos += nextcomma + 1 
2065                  if numargs-1 in argnums: 
2066                      if first_occurrence and notFirst: 
2067                          # just include last argument as it was if this is
 
2068                          # the first occurrence
 
2069                          output += source[currpos:endbrace+1] 
2070                      else: 
2071                          # last argument needs to wrapped too
 
2072                          argstr = source[currpos:endbrace] 
2073                          # get rid of leading or tailing whitespace
 
2074                          argstr = argstr.strip() 
2075                          output += wrapL + argstr + wrapR + ')' 
2076                      first_occurrence = False 
2077                  else: 
2078                      # just include last argument as it was
 
2079                      output += source[currpos:endbrace+1] 
2080              else: 
2081                  if argnums[0] != 0: 
2082                      raise ValueError("Specified argument number out of range") 
2083                  if first_occurrence and notFirst: 
2084                      output += source[currpos:endbrace+1] 
2085                  else: 
2086                      argstr = source[currpos:endbrace] 
2087                      # get rid of leading or tailing whitespace
 
2088                      argstr = argstr.strip() 
2089                      output += wrapL + argstr + wrapR + ')' 
2090                  first_occurrence = False 
2091              currpos = endbrace+1 
2092          else: 
2093              output += source[currpos:] 
2094      return output

2095  
 
2096  
 
2097  ##def replaceCallsWithDummies(source, callfns, used_dummies=None, notFirst=False):
 
2098  ##    """Replace all function calls in source with dummy names,
 
2099  ##    for the functions listed in callfns. Returns a pair (new_source, d)
 
2100  ##    where d is a dict mapping the dummy names used to the function calls."""
 
2101  ##    # This function used to work on lists of callfns directly, but I can't
 
2102  ##    # see why it stopped working. So I just added this recursing part at
 
2103  ##    # the front to reduce the problem to a singleton function name each time.
 
2104  ##    print "\nEntered with ", source, callfns, used_dummies
 
2105  ##    if used_dummies is None:
 
2106  ##        used_dummies = 0
 
2107  ##    if isinstance(callfns, list):
 
2108  ##        if len(callfns) > 1:
 
2109  ##            res = source
 
2110  ##            dummies = {}
 
2111  ##            #remaining_fns = callfns[:]
 
2112  ##            for f in callfns:
 
2113  ##                #remaining_fns.remove(f)
 
2114  ##                new_res, d = replaceCallsWithDummies(res, [f], used_dummies, notFirst)
 
2115  ##                res = new_res
 
2116  ##                if d != {}:
 
2117  ##                    dummies.update(d)
 
2118  ##                    used_dummies = max( (used_dummies, max(d.keys())) )
 
2119  ##            if dummies != {}: # and remaining_fns != []:
 
2120  ##                new_dummies = dummies.copy()
 
2121  ##                for k, v in dummies.items():
 
2122  ##                    new_v, new_d = replaceCallsWithDummies(v, callfns,
 
2123  ##                                                used_dummies, notFirst=True)
 
2124  ##                    new_dummies[k] = new_v
 
2125  ##                    if new_d != {}:
 
2126  ##                        new_dummies.update(new_d)
 
2127  ##                        used_dummies = max( (used_dummies, max(new_dummies.keys())) )
 
2128  ##                dummies.update(new_dummies)
 
2129  ##            return res, dummies
 
2130  ##    else:
 
2131  ##        raise TypeError("Invalid list of function names")
 
2132  ##    done = False
 
2133  ##    dummies = {}
 
2134  ##    output = ""
 
2135  ##    currpos = 0
 
2136  ##    doneFirst = False
 
2137  ##    while not done:
 
2138  ##        # find any callfns in source (now just always a singleton)
 
2139  ##        findposlist_candidates = [source[currpos:].find(fname+'(') for fname in callfns]
 
2140  ##        findposlist = []
 
2141  ##        for candidate_pos in findposlist_candidates:
 
2142  ##            # remove any that are actually only full funcname matches to longer strings
 
2143  ##            # that happen to have that funcname at the end: e.g. functions ['if','f']
 
2144  ##            # and find a match 'f(' in the string 'if('
 
2145  ##            if currpos+candidate_pos-1 >= 0:
 
2146  ##                if not isNameToken(source[currpos+candidate_pos-1]):
 
2147  ##                    findposlist.append(candidate_pos)
 
2148  ##            else:
 
2149  ##                # no earlier character in source, so must be OK
 
2150  ##                findposlist.append(candidate_pos)
 
2151  ##        if len(findposlist) > 0 and notFirst:
 
2152  ##            findposlist = findposlist[1:]
 
2153  ##        try:
 
2154  ##            findpos = min(filter(lambda x:x>=0, findposlist))+currpos
 
2155  ##        except ValueError:
 
2156  ##            done = True
 
2157  ##        if not done:
 
2158  ##            # find start and end braces
 
2159  ##            startbrace = source[findpos:].find('(')+findpos
 
2160  ##            endbrace = findEndBrace(source[startbrace:])+startbrace
 
2161  ##            sub_source = source[startbrace+1:endbrace]
 
2162  ##            embedded_calls = [sub_source.find(fname+'(') for fname in callfns]
 
2163  ##            try:
 
2164  ##                subpositions = filter(lambda x:x>0, embedded_calls)
 
2165  ##                if subpositions == []:
 
2166  ##                    filtered_sub_source = sub_source
 
2167  ##                    new_d = {}
 
2168  ##                else:
 
2169  ##                    filtered_sub_source, new_d = \
 
2170  ##                            replaceCallsWithDummies(sub_source,
 
2171  ##                                                    callfns, used_dummies)
 
2172  ##            except ValueError:
 
2173  ##                pass
 
2174  ##            else:
 
2175  ##                if new_d != {}:
 
2176  ##                    dummies.update(new_d)
 
2177  ##                    used_dummies = max( (used_dummies, max(dummies.keys())) )
 
2178  ##            used_dummies += 1
 
2179  ##            dummies[used_dummies] = source[findpos:startbrace+1] + \
 
2180  ##                                      filtered_sub_source + ')'
 
2181  ##            output += source[currpos:findpos] + '__dummy%i__' % used_dummies
 
2182  ##            currpos = endbrace+1
 
2183  ##        else:
 
2184  ##            output += source[currpos:]
 
2185  ##    return output, dummies
 
2186  
 
2187  
 


2188 -def replaceCallsWithDummies(source, callfns, used_dummies=None, notFirst=False):


2189      """Replace all function calls in source with dummy names,
 
2190      for the functions listed in callfns. Returns a pair (new_source, d)
 
2191      where d is a dict mapping the dummy names used to the function calls.
 
2192      """ 
2193      # This function used to work on lists of callfns directly, but I can't
 
2194      # see why it stopped working. So I just added this recursing part at
 
2195      # the front to reduce the problem to a singleton function name each time.
 
2196      if used_dummies is None: 
2197          used_dummies = 0 
2198      done = False 
2199      dummies = {} 
2200      output = "" 
2201      currpos = 0 
2202      doneFirst = False 
2203      while not done: 
2204          # find any callfns in source (now just always a singleton)
 
2205          findposlist_candidates = [source[currpos:].find(fname+'(') for fname in callfns] 
2206          findposlist = [] 
2207          for candidate_pos in findposlist_candidates: 
2208              # remove any that are actually only full funcname matches to longer strings
 
2209              # that happen to have that funcname at the end: e.g. functions ['if','f']
 
2210              # and find a match 'f(' in the string 'if('
 
2211              if currpos+candidate_pos-1 >= 0: 
2212                  if not isNameToken(source[currpos+candidate_pos-1]): 
2213                      findposlist.append(candidate_pos) 
2214              else: 
2215                  # no earlier character in source, so must be OK
 
2216                  findposlist.append(candidate_pos) 
2217          findposlist = [ix for ix in findposlist if ix >= 0] 
2218          findposlist.sort() 
2219          if not doneFirst and notFirst and len(findposlist) > 0: 
2220              findposlist = findposlist[1:] 
2221              doneFirst = True 
2222          try: 
2223              findpos = findposlist[0]+currpos 
2224          except IndexError: 
2225              done = True 
2226          if not done: 
2227              # find start and end braces
 
2228              startbrace = source[findpos:].find('(')+findpos 
2229              endbrace = findEndBrace(source[startbrace:])+startbrace 
2230              sub_source = source[startbrace+1:endbrace] 
2231              embedded_calls = [sub_source.find(fname+'(') for fname in callfns] 
2232              try: 
2233                  subpositions = filter(lambda x:x>0, embedded_calls) 
2234                  if subpositions == []: 
2235                      filtered_sub_source = sub_source 
2236                      new_d = {} 
2237                  else: 
2238                      filtered_sub_source, new_d = \
 
2239                              replaceCallsWithDummies(sub_source,
 
2240                                                      callfns, used_dummies) 
2241              except ValueError: 
2242                  pass 
2243              else: 
2244                  if new_d != {}: 
2245                      dummies.update(new_d) 
2246                      used_dummies = max( (used_dummies, max(dummies.keys())) ) 
2247              used_dummies += 1 
2248              dummies[used_dummies] = source[findpos:startbrace+1] + \
 
2249                                        filtered_sub_source + ')' 
2250              output += source[currpos:findpos] + '__dummy%i__' % used_dummies 
2251              currpos = endbrace+1 
2252          else: 
2253              output += source[currpos:] 
2254      if dummies != {}: 
2255          new_dummies = dummies.copy() 
2256          for k, v in dummies.items(): 
2257              new_v, new_d = replaceCallsWithDummies(v, callfns,
 
2258                                          used_dummies, notFirst=True) 
2259              new_dummies[k] = new_v 
2260              if new_d != {}: 
2261                  new_dummies.update(new_d) 
2262                  used_dummies = max( (used_dummies, max(new_dummies.keys())) ) 
2263          dummies.update(new_dummies) 
2264      return output, dummies

2265  
 
2266  
 


2267 -def addArgToCalls(source, callfns, arg, notFirst=''):


2268      """Add an argument to calls in source, to the functions listed in callfns.
 
2269      """ 
2270      # This function used to work on lists of callfns directly, but I can't
 
2271      # see why it stopped working. So I just added this recursing part at
 
2272      # the front to reduce the problem to a singleton function name each time.
 
2273      if isinstance(callfns, list): 
2274          if len(callfns) > 1: 
2275              res = source 
2276              for f in callfns: 
2277                  res = addArgToCalls(res, [f], arg, notFirst) 
2278              return res 
2279      else: 
2280          raise TypeError("Invalid list of function names") 
2281      done = False 
2282      output = "" 
2283      currpos = 0 
2284      while not done: 
2285          # find any callfns in source (now just always a singleton)
 
2286          findposlist_candidates = [source[currpos:].find(fname+'(') for fname in callfns] 
2287          findposlist = [] 
2288          for candidate_pos in findposlist_candidates: 
2289              # remove any that are actually only full funcname matches to longer strings
 
2290              # that happen to have that funcname at the end: e.g. functions ['if','f']
 
2291              # and find a match 'f(' in the string 'if('
 
2292              if currpos+candidate_pos-1 >= 0: 
2293                  if not isNameToken(source[currpos+candidate_pos-1]): 
2294                      findposlist.append(candidate_pos) 
2295              else: 
2296                  # no earlier character in source, so must be OK
 
2297                  findposlist.append(candidate_pos) 
2298          try: 
2299              findpos = min(filter(lambda x:x>=0,findposlist))+currpos 
2300          except ValueError: 
2301              if currpos < len(source) and len(findposlist_candidates) > 0 and \
 
2302                         findposlist_candidates[0] >= 0: 
2303                  currpos += findposlist_candidates[0] + 2 
2304                  output += source[:findposlist_candidates[0]+2] 
2305                  continue 
2306              else: 
2307                  done = True 
2308          if not done: 
2309              # find start and end braces
 
2310              startbrace = source[findpos:].find('(')+findpos 
2311              endbrace = findEndBrace(source[startbrace:])+startbrace 
2312              sub_source = source[startbrace+1:endbrace] 
2313              embedded_calls = [sub_source.find(fname+'(') for fname in callfns] 
2314              try: 
2315                  subpositions = filter(lambda x:x>0,embedded_calls) 
2316                  if subpositions == []: 
2317                      filtered_sub_source = sub_source 
2318                  else: 
2319                      filtered_sub_source = addArgToCalls(sub_source,callfns,arg,notFirst) 
2320              except ValueError: 
2321                  pass 
2322              # add unchanged part to output
 
2323              # insert arg before end brace
 
2324              if currpos==0 and callfns == [notFirst]: 
2325                  notFirst = '' 
2326                  addStr = '' 
2327              else: 
2328                  if filtered_sub_source == '': 
2329                      addStr = arg 
2330                  else: 
2331                      addStr = ', ' + arg 
2332              output += source[currpos:startbrace+1] + filtered_sub_source \
 
2333                      + addStr + ')' 
2334              currpos = endbrace+1 
2335          else: 
2336              output += source[currpos:] 
2337      return output

2338  
 
2339  
 


2340 -def proper_match(specstr, term):


2341      """Determine whether string argument 'term' appears one or more times in
 
2342      string argument 'specstr' as a proper symbol (not just as part of a longer
 
2343      symbol string or a number).
 
2344      """ 
2345      ix = 0 
2346      term_len = len(term) 
2347      while ix < len(specstr) and term_len > 0: 
2348          pos = specstr[ix:].find(term) + ix 
2349          if pos > -1: 
2350              try: 
2351                  if specstr[pos + term_len] not in [')', '+', '-', '/', '*', ' ',
 
2352                                            ']', ',', '<', '>', '=', '&', '^']: 
2353                      # then term continues with additional name characters:
 
2354                      # no match after all
 
2355                      ix = pos + term_len 
2356                      continue 
2357              except IndexError: 
2358                  # no other chars remaining, so doesn't matter
 
2359                  pass 
2360              if isNumericToken(term): 
2361                  # have to be careful that we don't replace part
 
2362                  # of another number, e.g. origterm == '0'
 
2363                  # with repterm == 'abc', and the numeric literal '130'
 
2364                  # appears in specstr. The danger is that we'd end up
 
2365                  # with new specstr = '13abc'
 
2366                  if specstr[pos-1] in num_chars + ['.', 'e'] or \
 
2367                     specstr[pos + term_len] in num_chars + ['.', 'e']: 
2368                      ix = pos + term_len 
2369                      continue 
2370              return True 
2371          else: 
2372              break 
2373      return False

2374  
 
2375  
 


2376 -def remove_indices_from_range(ixs, max_ix):


2377      """From the indices 0:max_ix+1, remove the individual
 
2378      index values in ixs.
 
2379      Returns the remaining ranges of indices and singletons.
 
2380      """ 
2381      ranges = [] 
2382      i0 = 0 
2383      for ix in ixs: 
2384          i1 = ix - 1 
2385          if i1 < i0: 
2386              i0 = ix + 1 
2387          elif i1 == i0: 
2388              ranges.append([i0]) 
2389              i0 = ix + 1 
2390          else: 
2391              ranges.append([i0,i1+1]) 
2392              i0 = ix + 1 
2393      if i0 < max_ix: 
2394          ranges.append([i0, max_ix+1]) 
2395      elif i0 == max_ix: 
2396          ranges.append([i0]) 
2397      return ranges

2398
```

  


| Home | Trees | Indices | Help | PyDSTool |
| --- | --- | --- | --- | --- |

|  |  |
| --- | --- |
| Generated by Epydoc 3.0.1 on Fri May 4 15:24:20 2012 | http://epydoc.sourceforge.net |
